# Measuring cardiac stroke volume through in-ear audio sensing

**DOI:** 10.1038/s41467-026-75642-0

**Published:** 2026-07-17

**Authors:** Kayla-Jade Butkow, Navazh Jalaludeen, Yang Liu, Jake Stuchbury-Wass, Qiang Yang, Mathias Ciliberto, Dong Ma, Joseph Cheriyan, Cecilia Mascolo

**Affiliations:** 1 Department of Computer Science and Technology, University of Cambridge, Cambridge, United Kingdom; 2 Clinical Pharmacology, Cambridge University Hospitals NHS Foundation Trust, Cambridge, United Kingdom; 3 Department of Medicine, Division of Experimental Medicine and Immunotherapeutics, Cambridge, United Kingdom; 4 Cardiovascular Trials Office, Cambridge Clinical Trials Unit, Cambridge University Hospitals NHS Foundation Trust, Cambridge, United Kingdom

**Keywords:** Biomedical engineering, Cardiology

## Abstract

Stroke volume, the volume of blood ejected by the left ventricle during a contraction, is a key metric of cardiovascular health. Currently, stroke volume is measured in clinic with specialised equipment. While purpose-made wearables exist to measure stroke volume, no solution relies solely on commodity devices. We present a deep learning system for stroke volume estimation from in-ear audio of earbuds. We combine generative self-supervised/transfer learning, a transformer-based autoencoder, to predict average stroke volume in unseen subjects. With data from 23 healthy participants, we compare our estimations to clinically validated device estimations. We achieve a mean absolute error of 5.24 ml, a Pearson correlation of r=0.94 between average predicted stroke volume and average true stroke volume, and a Percentage Error in the limits of agreement of 11.05% (within clinical range for stroke volume measurement devices). These findings open the doors to longitudinal, scalable and affordable cardiovascular measurement out of clinic.

## Abstract (web summary)

Stroke volume is a key indicator of cardiac function but requires specialised equipment to measure. Here the authors show that a deep learning model using in-ear audio from standard earbuds can accurately estimate stroke volume.

## Introduction

Stroke volume, the volume of blood ejected by the left ventricle in each contraction, is one of the fundamental measures of cardiovascular physiology. It is closely related to the cardiac output (CO), which represents the total volume of blood pumped by the heart per minute. Stroke volume is an important determinant of CO and provides an estimate of the body’s ability to deliver oxygen to the tissues (tissue perfusion) and its ventricular function^1^, making it crucial in evaluating the health of the circulatory system.

Stroke volume is a key parameter in sports medicine and exercise physiology, used to assess overall cardiovascular health and fitness. Higher stroke volumes are often observed in athletes with more efficient cardiovascular systems, making it an excellent indicator of cardiovascular fitness^2,3^. There is thus potential for stroke volume measurements to be used to monitor improvements in cardiovascular fitness over time due to exercise and to customise training programs to meet lifestyle and fitness goals. Moreover, stroke volume directly reflects the efficiency of the myocardium, the resistance to blood flow from the heart (afterload), and the return of blood to the heart (preload). When one of these is disrupted, the resulting stroke volume changes can serve as an early indicator of subclinical changes in cardiovascular function, potentially revealing abnormalities in healthy individuals before observable disease symptoms emerge. Stroke volume therefore has the potential to be used for prediction, early diagnosis and continuous monitoring of cardiovascular health in normal individuals. Clinically, low stroke volume can be a strong indicator of heart failure or other cardiac conditions, such as arrhythmias and heart valve diseases^4,5^. Therefore, understanding and measuring stroke volume is essential for evaluating cardiac function, which plays a critical role in diagnosing and managing cardiovascular diseases^5^ and monitoring the progression of disease state in patients with cardiac pathologies.

The gold standard for stroke volume measurement is the Direct Fick method, which involves using pulmonary arterial or transpulmonary catheters^6,7^. However, this method is highly invasive, requiring multiple catheters, and is very labour-intensive^8^. As a result, echocardiography (or cardiac ultrasound) has become more prevalent as a non-invasive alternative for calculating stroke volume^7^. Echocardiography uses ultrasound waves to generate images of the heart and its blood flow, and when combined with Doppler flow velocity recordings, it can be used to estimate stroke volume. However, this technique requires highly skilled operators to accurately determine stroke volume, limiting its widespread use^7^. Additionally, it is not suitable for continuous stroke volume monitoring. For non-invasive, continuous stroke volume measurements, finger-cuff systems, such as the Finapres NOVA^9^, can be used. These systems use an inflatable finger cuff and photoplethysmography (PPG) to determine arterial blood pressure and thus stroke volume. While reliable, these systems are cumbersome and require calibration using a stroke volume measurement from an echocardiogram^10^, limiting their utility. In summary, all of these methods only have clinical use due to their dependence on complex procedures, highly specialised and expensive equipment, and trained personnel. Additionally, these techniques have limited accessibility worldwide due to their high cost and limited scalability.

Wearable devices have emerged as an alternative for non-invasive stroke volume estimation outside of clinical settings. By enabling continuous monitoring of cardiovascular health, they can help detect deviations over time and potentially support early identification of cardiovascular disease. Around 2% of the adult population are estimated to have undiagnosed heart failure^11^, with misdiagnosis rates in general practice reaching up to 68%^12^. Continuous stroke volume assessment through wearables therefore offers an opportunity to detect early signs of pathophysiology, enabling earlier diagnosis and intervention. Beyond clinical use, these devices also provide value in tracking cardiovascular fitness across broader populations. Chest patches equipped with a single-lead electrocardiogram (ECG) and a triaxial accelerometer^13^ or with a PPG sensor^14^ have been used for CO and stroke volume measurements. However, these systems have a conspicuous and uncomfortable form factor, and often require calibration with a blood pressure cuff. Finger cuffs equipped with PPG have also been used^15^, though their bulky form factor limits real-world applicability. In addition, a bathroom weighing scale using ballistocardiography, electrocardiography, and impedance plethysmography has been developed to estimate stroke volume^6^. However, this device relies on specialised sensors not found in standard scales, requiring users to purchase a new device for monitoring. Additionally, it relies heavily on user adherence, limiting monitoring to the specific moments when the user steps on the scale, a well-known challenge with medical devices^16^. *Thus, there is currently no solution that provides continuous stroke volume monitoring in a convenient, unobtrusive, and widely accessible form factor using only commodity devices and their native sensors*.

Earables, sensor-equipped earbuds such as the Apple AirPods Pro or high-end hearing aids, have become increasingly pervasive in daily life. These commercial earables contain multiple on-board sensors, including outward-facing microphones for speech capture, and inward-facing microphones for active noise cancellation. Recent studies have shown that these in-ear microphones can also be leveraged to capture cardiac signals from inside the ear canal^17–20^. These cardiac signals, which contain fundamental information about events in the cardiac cycle (such as valve closures, contractions, and electrical stimulations)^20^, are related to stroke volume^10,13^. Additionally, studies have demonstrated a correlation between the amplitude of heart sounds collected on the chest and CO, and by extension, stroke volume^21^. Therefore, there is the potential to use in-ear heart sounds to infer stroke volume, presenting a unique opportunity to develop a commodity wearable-based method for stroke volume monitoring outside clinical settings.

In this work, we demonstrate the feasibility of estimating stroke volume using sensors on commodity earables in a robust, generalisable way. By capturing cardiac signals from inside the ear canal through sensors embedded in commodity earbuds, we present results suggesting the feasibility of affordable and scalable stroke volume estimation. To achieve this, we encounter three main challenges. Firstly, while relationships between various heart signal modalities and stroke volume have been identified, in-ear heart signals are complex and mapping them to stroke volume remains unclear and challenging. Secondly, as is common when developing new wearable technologies, collecting a large-size dataset is challenging due to the requirement for equipment and expertise, which hinders the development of high-performing algorithms. Finally, a key challenge in machine learning research is ensuring model generalisability, particularly when faced with data from unseen subjects with varying stroke volumes, and in-ear audio signal properties.

As a solution, we develop a two-stage training process that integrates generative self-supervised pre-training with fine-tuning. We introduce CardiAural, a pipeline designed for estimating stroke volume using in-ear audio. First, to address the initial challenge, we apply self-supervised learning techniques that mask and reconstruct intricate in-ear heart signals. Training the model to reconstruct randomly masked spectrogram representations allows it to learn complex and robust features crucial for accurate stroke volume estimation. Second, to tackle the issue of data scarcity, we utilise an in-house dataset of unlabelled earable data for self-supervised pre-training. This enables the model to acquire generalised and resilient representations of in-ear audio without the necessity for large labelled datasets. Finally, we fine-tune the model with a smaller, highly specialised dataset specifically collected for stroke volume estimation. This two-stage approach not only addresses data scarcity but also enhances the model’s ability to generalise effectively.

To evaluate the performance of CardiAural, we collected in-ear audio using a custom earbud device. We compare our stroke volume estimations to those obtained with a clinically validated device in a cohort of healthy participants (*N* = 23) with an average weight ($\bar{x}\pm \sigma$) of 76 ± 15kg and an average height of 158 ± 9cm, and assess performance before and after isometric exercise. Overall, we achieve a Mean Absolute Percentage Error of 6.83% in estimating the average stroke volume of unseen subjects by training the model on data from all other participants. This demonstrates the model’s strong ability to predict stroke volume accurately, even for unseen participants. Additionally, we achieve a percentage error in the limits of agreement of 11.05%, which is well below the 30% error considered acceptable for a stroke volume measurement device^22^, and which outperforms all wearable-based systems in literature. This effectively indicates that commodity earables can be a suitable stroke volume measurement tool. More specifically, our findings highlight the feasibility of using a single sensor on a commodity earbud to obtain accurate stroke volume measurements, paving the way for out-of-the-clinic cardiac monitoring using wearable devices.

## Results

In this section, we present the results of stroke volume estimation using in-ear audio. First, we showcase in-ear audio signals and their corresponding stroke volume measurements to highlight the feasibility of our approach, followed by a presentation of the estimation ability of our system.

### Correlation between in-ear heart signals and stroke volume

**Fig. 1:**
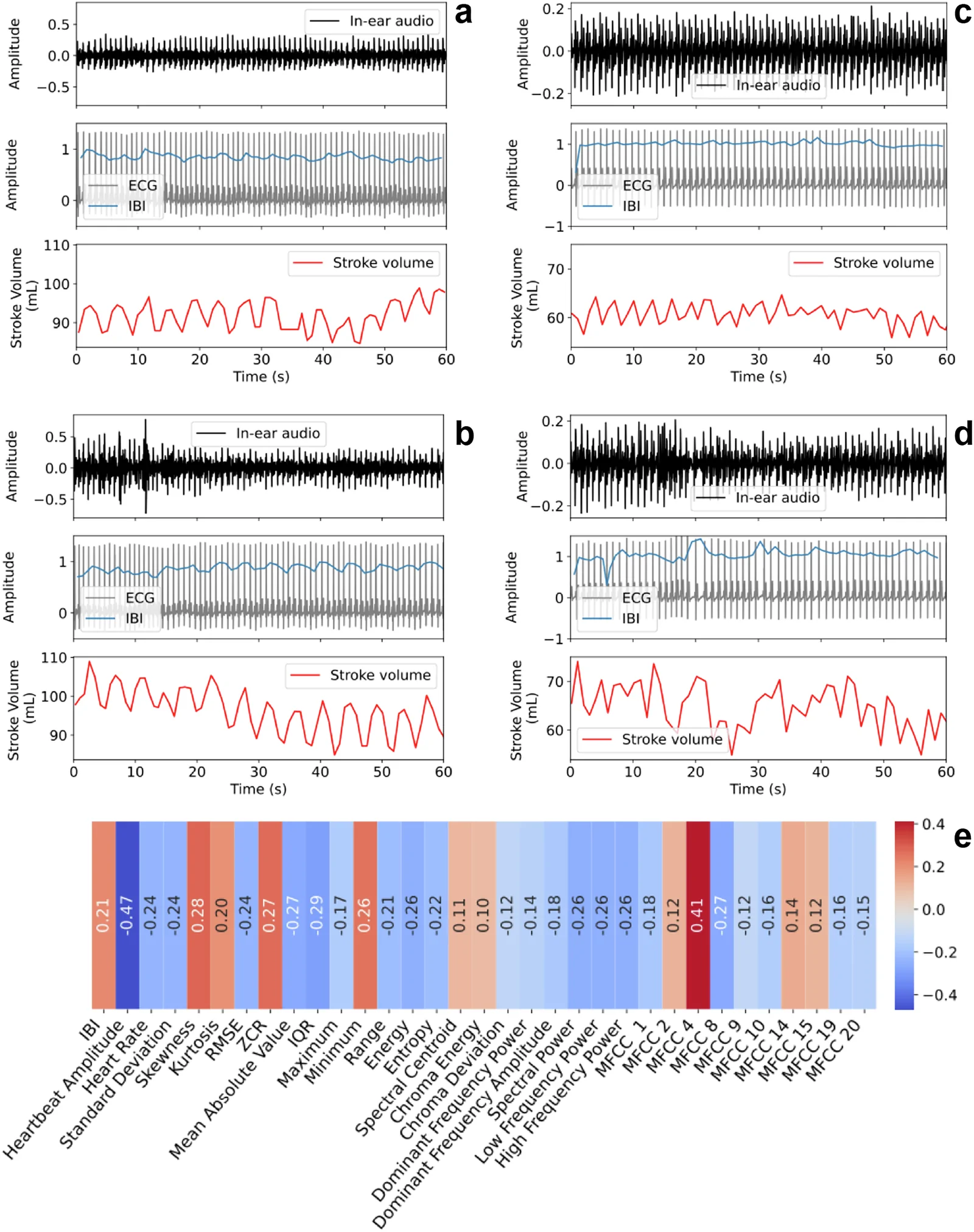
Signal properties of the in-ear audio, ECG, stroke volume signals, and correlations between the in-ear audio signal and stroke volume. **a**–**d** 60 s of in-ear audio, ECG, inter-beat interval (IBI) and stroke volume for two participants before and after isometric exercise. **a**, **b** Participant 1 before and after exercise, respectively. **c**, **d** Participant 2 before and after exercise, respectively. In-ear audio exhibits consistent heart signals, with a peak corresponding to each heartbeat in the ECG signal. Before exercise, stroke volume maintains a consistent level. In the after-exercise condition, stroke volume is elevated and slowly returns to baseline levels. **e** Correlations between features extracted from the in-ear audio and the stroke volume. Weak correlations exist between heart-related properties and stroke volume, and between audio features and stroke volume. This highlights the potential of using in-ear audio to estimate stroke volume and emphasises the need for advanced learning techniques to intelligently leverage the weak correlations.

We present an example of ground truth stroke volume collected from two participants and their corresponding in-ear audio in Fig. 1. The corresponding ECG signals and their interbeat intervals (IBIs) are also collected to validate the effectiveness of in-ear audio for capturing cardiac activity. Figure 1a, b presents the stroke volume for one participant before and after isometric exercise, respectively, and Fig. 1c, d presents the same for a second participant. For each participant, 60 s of data per condition is shown. For Participant 1, the mean stroke volume in the 60 s was 91 ml before exercise and 96 mL afterwards, with an average heart rate of 70 BPM. For Participant 2, the mean stroke volumes were 60 ml and 65 ml, respectively, with a heart rate of 61 BPM. The two participants are the same age, with different fitness levels (as deduced from their exercise frequency), which is reflected in the different stroke volumes. Moreover, the alignment of the peaks of the in-ear heart signals and the ECG shows the correlation between the two signals. This provides evidence that the variations in the in-ear signals are due to heart activity.

Immediately following isometric exercise, both participants exhibit an increase in the amplitude of the in-ear audio signal (Fig. 1b, d). This increase corresponds with elevated stroke volumes, which is consistent with expected physiological response to exercise. As the participants return to rest, the stroke volume gradually decreases, and this trend is mirrored by a reduction in the in-ear audio amplitude and a return of the IBI to baseline, indicating a lowering of heart rate and cardiac demand. In contrast, during the resting condition (Fig. 1a, c), the stroke volume remains relatively stable, and the in-ear waveform shows minimal variation over time.

This example illustrates that the in-ear signal reflects changes in stroke volume, not just due to heart rate but also due to beat-to-beat variability in cardiac output, as shown in the IBI and SV signals.

We further extracted common features of audio signals^23^, including signal statistics, time domain and frequency domain features, and features of the heart signal, including inter-beat interval (IBI), heart rate and the amplitude of the peak of the in-ear heart signal for each heartbeat. We then computed the Pearson correlation between the audio features in a 10-s window and the stroke volume for that window, removed any features with a correlation of less than ±0.1, and plotted the results. We explore this relationship between stroke volume and features of the in-ear audio in Fig. 1e. There is a moderate negative correlation between the amplitude of the largest peak in the in-ear audio signal per heartbeat and stroke volume, and between heart rate and stroke volume (heart-related features). There is also a moderate positive correlation between the skewness, zero crossing rate, the fourth Mel-Frequency Cepstral Coefficients (MFCC) component^23^ and the stroke volume (audio-related features). Other features exhibit weak positive and negative correlations. In summary, weak correlations exist between stroke volume and audio features, as well as between stroke volume and heart-related properties. This shows that in-ear audio has the potential to estimate stroke volume, however, handcrafted features alone struggle to generalise to differing signal properties. Later in this section, we provide some evidence of this limited generalisability to contextualise the predictive power of these features. Deep learning models have an inherent ability to approximate complex relationships and identify intricate patterns and features from data, making them a natural choice to solve this challenge.

Additionally, the lack of generalisability of features is clear by comparing the in-ear audio signals in Fig. 1a–d. It is clear that the amplitude range of the in-ear audio of the two participants differs and the signals present distinct properties per person. Specifically, comparing common metrics of signal characteristics, the root mean square energy (RMSE) of the in-ear audio signal from Participant 1 is 19.98, with a skewness of 0.28, kurtosis of 2.38 and zero-crossing rate (ZCR) of 0.025. In contrast, for Participant 2, the RMSE is 11.30, with a skewness of 0.13, kurtosis of 0.72 and ZCR of 0.019. Thus, the signals exhibit large variability between participants, which weakens the correlations in Fig. 1e and makes generalisation to different users challenging. It is therefore necessary to develop techniques to adapt to this inter-participant signal variability. We solve this by using a generative self-supervised learning framework with a masked autoencoder, and present the estimation results in the coming sections.

### Overall stroke volume prediction

**Fig. 2:**
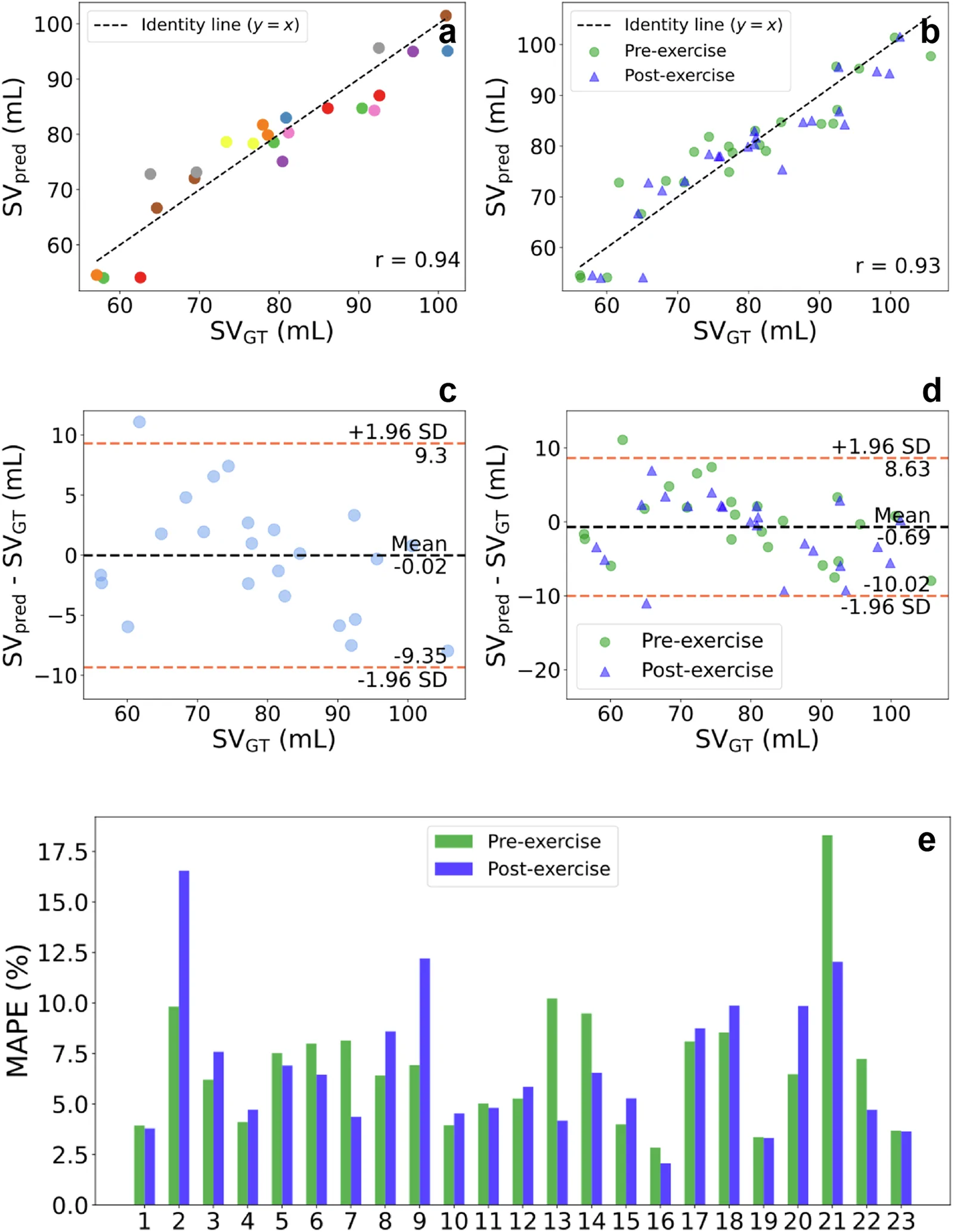
Overall stroke volume estimation results. **a** Scatter plot comparing the overall calculated stroke volume (SV_pred_) and the ground truth stroke volume (SV_GT_) per user. The Pearson correlation coefficient *r* quantifies the quality of correlation. **b** Relationship between the calculated stroke volume and the ground truth stroke volume for the two exercise conditions: before exercise (green circles) and after exercise (blue triangles). **c** Bland–Altman plot indicating the agreement of the overall estimated stroke volume with the overall ground truth stroke volume per participant. The horizontal lines indicate the mean value of the difference and the 95% limits of agreement (*d* and *d* ± 1.96*σ*). **d** Bland–Altman plot showing the agreement between the calculated and ground truth stroke volumes over the exercise conditions for each participant. Markers correspond to the two exercise conditions: before exercise (green circles) and after exercise (blue triangles). **e** Per-participant estimation mean absolute percentage error (MAPE) for the two conditions.

We now present the results of our deep learning-based system for stroke volume estimation from in-ear audio. Overall, our system can predict stroke volume from in-ear audio signals for unseen subjects with a mean absolute error (MAE) of 5.24 ml, a mean absolute percentage error (MAPE) of 6.83% and 95% limits of agreement of −9.35 ml to 9.31 ml when compared to the clinical standard Finapres Nova. In Fig. 2a, we provide the scatter plot of the stroke volume predictions per subject, including the identity line (the ideal line where predictions are equal to the ground truth). This plot examines the average stroke volume per subject over the entire experimental protocol. The calculated stroke volume is correlated with the ground truth stroke volume with a Pearson’s correlation coefficient (r) of 0.94 (*p* < 0.001), indicating a very high agreement between the predictions and the ground truth. The *R*^2^ score of 0.88 indicates that 88% of the variance in the ground truth stroke volume is explained by the predictions, indicating a close alignment between the predictions and the true values. From Fig. 2a, it appears that, generally, stroke volume estimations in the centre of the range are highly accurate. Towards the ends of the range, there is more variability with a slight tendency to underestimate stroke volume. Nonetheless, the system predicts average stroke volume, even for new users, with excellent agreement with the ground truth as indicated by both the low error and the high correlation coefficient. Figure 2c provides a Bland–Altman analysis of the overall average stroke volume per participant. From this plot, it is clear that one participant is an outlier in the system, achieving an error that exceeds the limits of agreement of the Bland–Altman plot. However, generally, the agreement between the measurements and the ground truth is good.

If we group the participants by sex, we achieve a MAE of 5.02 ml for females and 5.34 ml for males. Using an independent *T*-test, the difference is not statistically significant, highlighting the generalisability of our system.

The overall percentage error (PE) is 11.05%, which is well below the 30% PE deemed acceptable for a new stroke volume measurement device by Critchley and Critchley^24^, highlighting the potential of our solution. To further contextualise this result, we compare our performance with the reported results of other wearable devices used in literature in Table 1. Our system outperforms all prior works, with significantly lower PE, while additionally using only commodity sensors that are readily available in commercial devices. However, it is important to note that all previous studies used different ground truth devices for comparison, implying that there is potential for bias in the results due to inherent bias in the ground truth measurement device. Specifically, the Direct Fick method used by Yadzi et al.^6^ is the clinical gold-standard for stroke volume estimation, and the standard for which Critchley and Critchley’s^24^ 30% PE is devised. Thus, although our results look promising and the Finapres has been shown to have a high agreement with echocardiogram derived stroke volume, further work is required to compare our estimations to measurements from the Direct Fick method to assess clinical suitability.

**Table 1:** Performance comparison between our system and wearable-based systems reported in the literature

Author	Modality	Percentage error (%)	Ground truth method
Ours	Earable	**11.05**	FinaPres NOVA
Ganti et al.^13^	Chest patch with a single-lead electrocardiogram and a triaxial accelerometer	28	Cardiac MRI
Dvir et al.^14^	Chest patch with PPG sensor	25.6	Pulse Contour Cardiac Output
Yadzi et al.^6^	Custom sensor embedded scale	36.73	Direct Fick Method
Wang et al.^15^	Finger-based PPG	16.2	Physio Flow PF-05 impedance cardiograph

Our results are highlighted in bold.

When testing the system using a 70–30 cross-validation, whereby 70% of all users’ data is used for training and the remaining 30% of the data is used for testing, we are able to achieve an error of 2.6 ml ± 1.88 ml (3.57%) in estimating the overall average stroke volume for a participant. This shows that there is very low error and variance when seen data is used for model training.

### Sensitivity to changes pre and post-exercise

Our experimental protocol involves recording data in a baseline resting state (pre-exercise), and then in a post-exercise state to assess the body’s response to exercise. The exercise condition, which occurs between these two assessments, involves isometric exercise designed to produce cardiovascular stress which alters stroke volume. Clinically, assessing stroke volume before and after exercise is crucial for evaluating a patient’s cardiovascular health and response to exercise. It is therefore necessary to evaluate whether the system can predict both changes in stroke volume due to exercise and the resting, stable state where significant changes in stroke volume are unlikely. Figure 2b provides a scatter plot of the stroke volume predictions per user per condition. The overall correlation between the ground truth stroke volume and calculated stroke volume of the pre-exercise and post-exercise conditions is 0.93 (*p* < 0.001) with an *R*^2^ score of 0.87, similar to that of the overall predictions. From the Bland–Altman analysis in Fig. 2d, we see that we achieve a bias of −0.69 ml and limits of agreement of −10.02 ml to 8.63 ml in the combined condition. The very low bias and narrow limits of agreement (which contain most of the data points) again indicate a strong agreement between the predictions and the measurements. However, the negative bias shows a slight tendency to underestimate, as was observed in Fig. 2a. There is no evidence of a difference in pattern between the pre and post-exercise conditions, which suggests that the model’s performance is consistent across the two conditions. From the plot, we compute the overall PE as 11.97%, again lower than 30%.

Separating the pre and post-exercise conditions, a correlation of 0.94 (*p* < 0.001) before exercise, and 0.93 (*p* < 0.001) after exercise is achieved. Likewise, before exercise, there is a bias of −0.02 ml and limits of agreement of −9.35 ml to 9.31 ml, while after exercise, the bias is −1.37 ml and limits of agreement are −10.50 ml to 7.77 ml. This shows that the system underestimates more in the post-exercise condition where stroke volumes are elevated. However, this tendency is minor and can be overcome in future by collecting more data with elevated stroke volumes by including longer exercise durations. From the bias and limits of agreement, our system achieves a PE of 12.06% and 11.65% for the pre and post-exercise conditions, respectively. The system achieves an MAE (MAPE) of 5.27 ml (6.81%) and 5.21 ml (6.85%) before and after exercise, respectively. The similarity in errors demonstrates the system’s ability to accurately predict stroke volume under both elevated volumes post-exercise and resting conditions, proving the system’s generalisability in predicting a range of stroke volume responses. This is essential for identifying changes in stroke volume patterns over time for managing health.

Moreover, the estimation error per participant for the pre and post-exercise conditions is provided in Fig. 2e. Overall, only two participants (2 and 21) have an MAPE of over 10%, highlighting the generalisability of our system. If we separate the two conditions, most users have an MAPE of less than 10%, showing that predictions are accurate for both conditions. However, for four participants, errors of over 10% are seen for one or both conditions. For participant 2, the high MAPE is due to a slight under-prediction of the post-exercise condition, which manifests as a large error due to the low ground truth value. Likewise, for participant 9, the pre-exercise stroke volume is accurately predicted but underestimated after exercise. In participant 13, the pre-exercise stroke volume is over-estimated. Participant 21 has the highest overall error of all participants with errors of above 10% in both the pre and post-exercise conditions. This is due to the participant having a low ground truth stroke volume which is over-estimated leading to a larger percentage error. However, the performance is excellent for the majority of users.

### Stroke volume estimation using demographics and audio features

Height, weight, body surface area and body mass index have been proven to be moderately correlated to stroke volume^25,26^. To validate the strength of our technique, we ran a study to predict the mean stroke volume per user using only their demographics (age, weight, height, and sex). Using a support vector regressor with demographics alone as features, stroke volume can be estimated with a MAE of 11.24 ml (15% error), with a correlation of 0.40 (*p* = 0.44). Thus, although there is a moderate correlation between the predictions and the labels, it is not statistically significant, indicating no evidence of a linear relationship between the two. Consequently, demographics alone cannot be used to estimate stroke volume.

Heart rate has been shown to have a high feature importance for stroke volume predictions^13^. As such, we use average heart rate to predict average stroke volume, achieving an MAE of 10.72 ml with an error of 14% (r = 0.2, not statistically significant), showing that heart rate is slightly more predictive of stroke volume than demographics, yet both achieve poor performance.

We further attempted to predict stroke volume within 10 s windows using the features extracted in Fig. 1e combined with the demographics. We compute the average predicted stroke volume and compare it to the average ground truth stroke volume. Using the best-performing regressor (the support vector regressor with radial basis function kernel), we achieve similar results with an MAE of 11.82 ml (15% error). Thus, this study confirms that feature extraction alone cannot achieve generalisable results on unseen users, and confirms the need for a more sophisticated system, as implemented in this work.

## Discussion

We have presented an approach for estimating stroke volume using in-ear audio captured by earables. By evaluating against a clinically validated measurement device, our investigation proves that heart signals captured using in-ear audio on earables can estimate a person’s baseline stroke volume even for new, unseen subjects. With an overall MAPE of 6.83% and a strong agreement (r = 0.94, *R*^2^ = 0.88) with ground truth measurements, our method not only meets but exceeds the standards for accurate stroke volume monitoring devices^22^. This excellent performance proves the feasibility of stroke volume monitoring using commodity in-ear microphones commonly found in earables.

By leveraging earables, which are becoming increasingly ubiquitous, our solution makes stroke volume monitoring accessible to a broad population. Additionally, using wearable devices lowers the cost barrier associated with medical equipment, making monitoring more feasible for a wider population. This democratisation of health technology could lead to earlier detection of cardiovascular issues and improved public health outcomes. Given that earables, such as earbuds and hearing aids, are already worn for extended periods throughout the day as part of daily routines, they are uniquely positioned to enable long-term and continuous tracking of stroke volume to monitor trends over time, allowing for the identification of subtle changes and long-term patterns in cardiac function to provide valuable insights for proactive health management. Additionally, this natural integration into users’ lives makes them an ideal platform for longitudinal monitoring, without requiring additional effort or changes in behaviour, thereby enhancing user compliance. The integration of earables into daily routines also enables stroke volume monitoring in real-world settings, which is likely to provide a more comprehensive view of cardiovascular health across different daily activities and scenarios.

While our study shows promising results, there are some limitations. Despite the dataset covering a wide range of stroke volumes, the overall standard deviation is only 15 ml. For comparison, a previous study involving 1450 individuals reported a standard deviation of 18 ml ^27^, indicating that our dataset reflects similar variability to that seen in a larger population. However, from a processing standpoint, a narrower standard deviation means less variability in the dataset, which may limit the model’s ability to generalise to stroke volumes further from the mean. This challenge is compounded by the diversity in the in-ear audio features, making it more difficult to accurately predict stroke volumes at the extremes of the range. This trend is evident in Fig. 2a, where the smallest estimation errors are found for users with stroke volumes near the dataset average, while errors tend to increase slightly towards the lower and upper ends of the range. Nonetheless, the results demonstrate that our system, utilising self-supervised learning, is robust and effective overall. To further improve the system’s generalisability, future work should focus on expanding the dataset to include a wider distribution of stroke volumes, ages, ethnic groups, and fitness levels, to enhance the model’s ability to generalise and minimise the risk of bias. Future work should also focus on collecting data during exercise to fully assess the ability of the system to predict the stroke volume response to exercise. Importantly, this expanded dataset will be integrated into our current framework without the need for changes to the already highly effective modelling technique.

Our current experimental protocol includes an isometric squeezing test (section “Sensitivity to changes pre and post-exercise”) and we demonstrate the change in stroke volume between pre and post exercise. Our future tests will include comparative analysis with other activities to widen the range of stroke volumes and the variability in the dataset. In particular we aim to compare the effect of cycling and running as this has been studied in the literature^28^.

This paper presents a feasibility study into audio-based stroke volume estimation, and as such, the study cohort includes a limited number of individuals. Although our participant pool has a similar size to the other studies cited in this work, we acknowledge that the size of the cohort might have influenced the results, because of the limited variance. Thus, a fair comparison between our work and other studies from the literature is difficult. We also highlight that we have conducted the study with a cohort of healthy individuals: we expect that the variance would be higher in a mixed cohort (control and patients), which might impact the results. Additionally, we acknowledge that while the Finapres NOVA is FDA-approved for stroke volume monitoring, it is not the gold-standard clinical method for such measurements. However, we believe that its use is appropriate to validate our findings on a healthy population where invasive catheters, as used in the gold-standard technique, are inappropriate.

Future work should also include integrating our system into commercial earbuds. Given that these devices already contain an inward-facing microphone, our software could be easily incorporated, thus facilitating the widespread adoption of stroke volume monitoring on commodity wearables. Once this has been done, longitudinal and large-scale epidemiological studies can be carried out to assess the long-term accuracy and reliability of our system, as well as investigate its effectiveness for monitoring patients with known cardiovascular diseases.

Overall, the implications of this technology extend into many aspects of life. Clinically, our system could provide physicians with a tool for at-home patient monitoring and telemedicine, insights into the diagnosis and monitoring of disease states where cardiac output may vary including heart failure, post-heart attack and overall fitness in other associated conditions e.g., in respiratory disease. It can also be used to continuously track a person’s stroke volume thereby detecting subtle changes over time in healthy individuals. For general well-being, it could be used to monitor improvements in cardiovascular health over time as a result of exercise and to develop personalised training programs aimed at enhancing performance. Therefore, our work represents a step towards making advanced cardiovascular monitoring accessible, affordable, and integrated into daily life using commodity wearables.

## Methods

### In-ear heart signals

When the ear canal is blocked, *e.g*.,by wearing earbuds, creating a seal with the canal, the occlusion effect occurs^19,20^. With this effect, the ear canal is turned into a low-pass filter, resulting in low-frequency signals (e.g., 0–100 Hz) within the ear canal being amplified. This amplification has a magnitude of up to 40dB in low frequencies. Within these low frequencies, biosignals, such as heart signals, can be captured using a microphone placed inside the ear canal^17,19^. Early works^19,20^ viewed these signals as heart sounds, attributed to the turbulent flow of blood in nearby vasculature. However, recent studies^29^ suggest that these signals are more closely related to changes in local pressure due to pressure waves from heartbeats. Regardless, all literature agrees that these signals relate to heart activity as validated in Fig. 1.

While the precise origin of these in-ear heart signals – whether heart sounds or pressure waves – remains unclear, cardiac signals of various kinds have been successfully used to estimate stroke volume, which motivates our study. For example, studies^6^ have utilised features from ballistocardiography on a weighing scale, while others^13^ have used features of seismocardiography signals on a chest patch for stroke volume determination. Both types of sensors are sensitive to body vibrations caused by heart activity, similar to the in-ear signals, which are related to ear canal vibrations. Furthermore, research leveraging heart sound features^10,21^ collected using stethoscopes, and studies using arterial pressure waveforms^30^ have demonstrated success in estimating stroke volume. Therefore, regardless of whether the in-ear signals are heart sounds or pressure waves, their strong correlation with cardiac activity suggests that they could be effectively used for stroke volume estimation.

### Custom wearable device

**Fig. 3:**
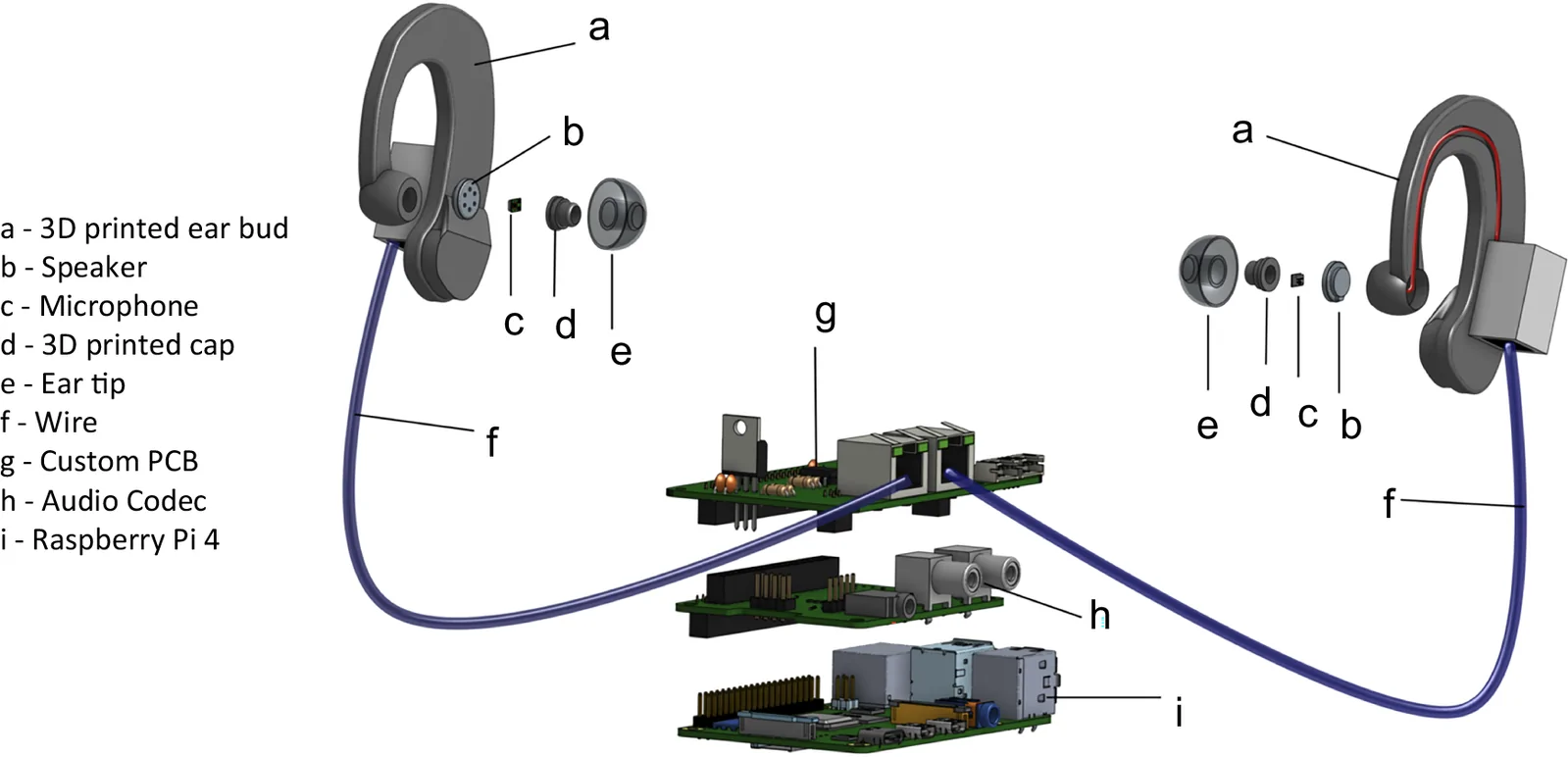
Exploded schematic of the custom earable system for in-ear audio collection. **a** Custom 3D printed earhook based on the design of Röddiger et al.^48^. **b** Speaker (a standard true wireless stereo 8mm, 16 Ohm earbud speaker) **c** Knowles SPU1410LR5H-QB microphone (**d**) 3D printed front cap (**e**) rubber ear tip (**f**) wire (**g**) custom PCB containing one MCP6004 non-inverting operational amplifier per channel (earbud). These amplifiers are controlled by potentiometers, allowing for adjustable gain if required. The gain value was set to unity for all data collection exercises. The PCB was designed to interface with the audio codec and relays the amplified microphone signals to the analog inputs of the codec. **h** HiFiBerry DAC+ ADC pro audio codec (**i**) Raspberry Pi 4. *Figure of the audio codec from* HiFiBerry 3D models of HiFiBerry boards (HiFiBerry), and model of the Raspberry Pi 4 from GrabCAD (Raspberry Pi 4 Model B, user-uploaded CAD model).

While in-ear microphones are becoming ubiquitous in active noise cancellation (ANC) earbuds^31,32^, no commercial earbud currently provides access to the raw data. To address this, we developed a custom earbud to collect in-ear heart signals, as shown in Fig. 3. Each earbud contains a small analogue microphone (Knowles SPU1410LR5H-QB) with a flat frequency response at low frequencies (10–100 Hz), enabling the capture of low-frequency in-ear heart sounds between 10 and 100 Hz. The microphone is secured inside a 3D-printed earbud, ensuring proper placement within the ear canal. To record the audio, we designed a PCB which interfaces with an audio codec^33^ on a Raspberry Pi 4. For portability, the system is powered by a portable power bank. We note that while we had to develop our own hardware prototype to prove our concepts, our algorithms are independent of this hardware and would be able to run on any commercial earbuds providing noise-cancelling functionality. To achieve the seal necessary for good performance, the user will be required to undergo an earbud fit test (as is implemented on the Apple AirPods Pro) to pick the eartips that fit their ears best.

### Dataset and collection protocol

A total of 23 healthy volunteers (detailed in Table 2) were recruited for this study, which aimed to evaluate the feasibility of determining stroke volume using in-ear audio. The study was approved by the Ethics Committee of the Department of Computer Science and Technology at the University of Cambridge, where data collection was conducted. Written informed consent was obtained from each participant prior to the start of the data collection protocol.

**Table 2:** Demographics of the study participants

Demographic	Mean	Min	Max	SD
Age	32	21	58	11
Weight (kg)	76	55	113	15
Height (cm)	176	158	194	9
Body mass index	25	19	42	5
Heart rate (BPM)	69	46	108	9
Stroke volume (ml)	79	45	118	15
Cardiac output (l)	5.41	2.97	11.06	1.09

**Fig. 4:**
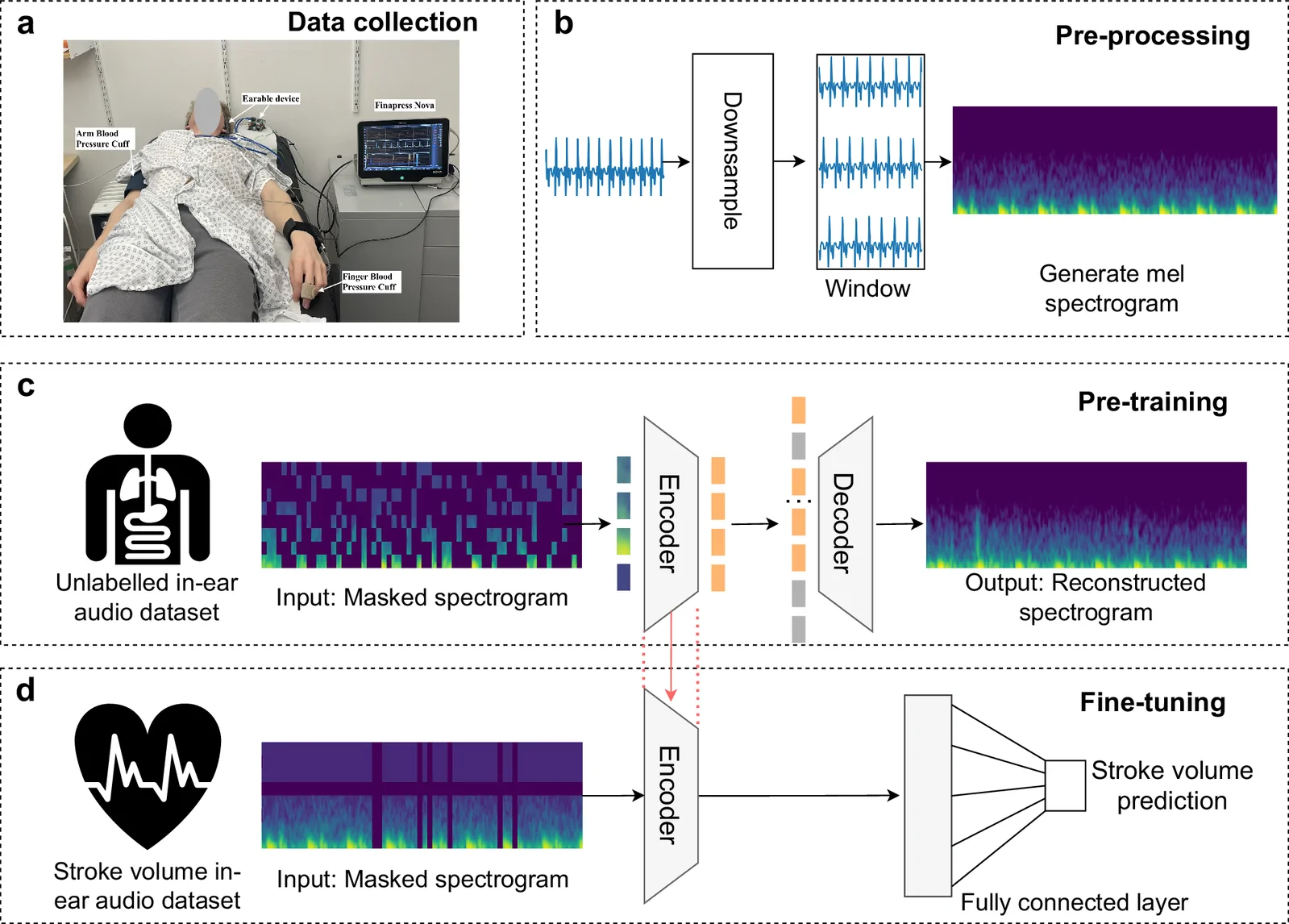
System processing pipeline. **a** Data collection protocol whereby data is simultaneously collected from our earable prototype and the ground truth using the Finapres NOVA continuous stroke volume measurement device. Not pictured is the isometric exercise where the participant squeezes a stress ball. **b** The pre-processing pipeline for the audio signals involves downsampling, segmenting the audio into 10-second windows with 8-second overlap, and generating a spectrogram for each window. **c** An unlabelled dataset of in-ear audio is used to pre-train the encoder-decoder network with masked spectrograms, employing a random, unstructured masking strategy. **d** The pretrained encoder is fine-tuned using the stroke volume in-ear audio dataset to predict stroke volume per 10-second window. During fine-tuning, a structured 2D masking strategy is used. Icon in (**c**): Internal Medicine, The Noun Project. Licensed under CC0 1.0 (public domain). Source: (Wikimedia Commons). Icon in (**d**): Cardiology, The Noun Project. Licensed under CC0 1.0 (public domain). Source: (Wikimedia Commons).

The study was conducted by trained medical professionals. The data collection process, illustrated in Fig. 4a, began with participants resting for 10 min on a bed. After this rest period, they performed isometric exercises, specifically squeezing a stress ball with quick, forceful contractions for 30 s, to elevate heart rate and alter stroke volume. Following the exercises, participants lay still for an additional 5 min. This protocol was designed to capture stroke volume measurements in the resting, baseline state, and in the exercise recovery state to evaluate the accuracy of our method under different conditions. In total, we collected 16 min of data per participant, and we analyse the data collected in the resting state and the recovery state.

During the data collection, subjects wore custom earbuds (Fig. 3) in both ears to collect in-ear audio signals, fitted with eartips of varying sizes to ensure proper occlusion. Ground truth stroke volume measurements were obtained using the Finapres NOVA continuous hemodynamic monitoring system^9^, which estimates beat-to-beat stroke volume through a PPG finger cuff.

The Finapres NOVA was selected for a number of reasons: Firstly, although the gold standard for measuring stroke volume is using thermodilution (direct Fick method), this involves an invasive catheter placed in the pulmonary artery, and is mainly used in critical care settings. Since we conducted this study on a healthy population, it was not ethically possible to use this technique. Secondly, although cardiac magnetic resonance imaging could be employed to measure stroke volume, there are a number of contraindications (presence of metal devices, renal dysfunction, contrast allergy, claustrophobia) and the utility of performing exercise whilst in an MRI machine is very limited. Additionally, concurrent use of a metallic earbud is contraindicated within an MRI. Therefore, we employed the use of a non-invasive device such as Finapres since it is one of only a few devices used in clinical research to assess beat-to-beat stroke volume.

However, in order to have an equivalent gold standard device for comparison for our study, we used non-invasive 2D echocardiography. This has previously been compared to invasive thermodilution in a meta-analysis^34^ of the two methods. This study reported a median bias of −0.12 L/min with limits of agreement ±0.94 L/min, and a median correlation coefficient of 0.827 (range: 0.140–0.998), indicating strong agreement between the two methods. The paper concluded that the two were interchangeable. Similarly, a study in cardiac patients^35^ found a high correlation (r = 0.91) and an average stroke volume difference of ~5 ml between echocardiography and the Fick method, further validating echocardiography as a reliable reference for calibration.

The FDA approval of Finapres mentions that the device should be calibrated to ensure accuracy. As such, we used echocardiogram as the best non-invasive gold standard calibration method for the Finapres in our outpatient, healthy volunteer study. Stroke volume was calculated using the GE Vivid IQ ultrasound system as the product of the left ventricular outflow tract (LVOT) cross-sectional area and the Doppler flow envelope recorded at the LVOT^7^. The average of three cardiac cycles was used. Once calibrated, when participants are lying down, Finapres NOVA has a mean bias of 0% ± 4.2% compared to echocardiogram^36^. There is thus good agreement between the two devices, meaning that our results experience minimal bias compared to ground truth readings taken from echocardiogram.

To set up the Finapres, a medical-grade three-lead ECG electrode was connected to each participant’s chest, and a blood pressure cuff was placed on their non-dominant arm for calibration. The system was calibrated using each participant’s stroke volume measurements from an echocardiogram. The Finapres was also calibrated using resting blood pressure measurements from the arm, taken while participants were lying down.

In-ear audio data was collected with a sampling rate of 44 kHz, while Finapres data was recorded on a beat-by-beat basis. The data streams were synchronised using timestamps recorded on both devices.

### Study population

**Fig. 5:**
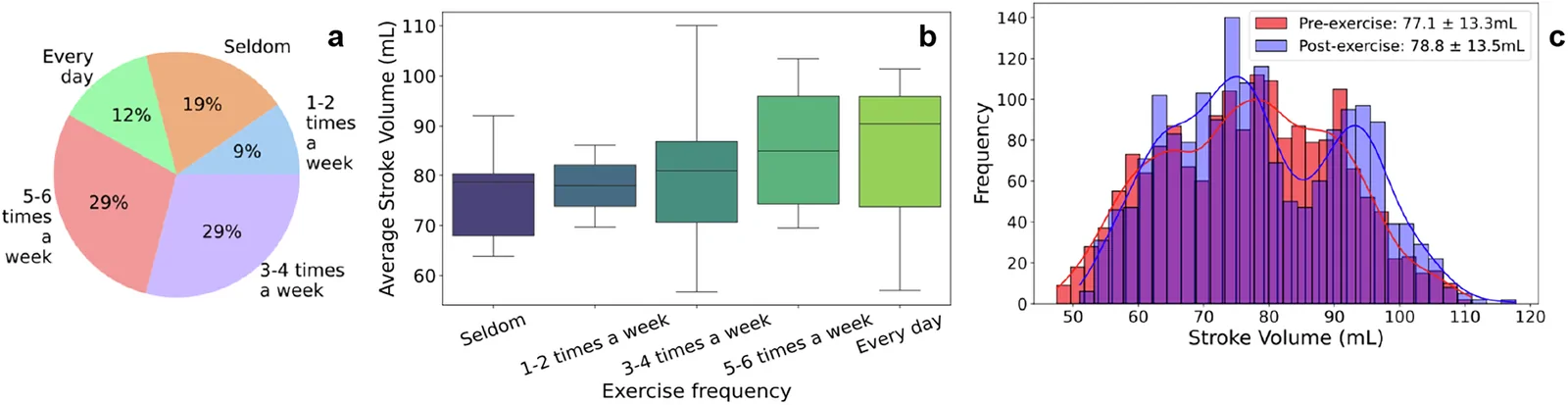
Overview of the impact of exercise on the participants’ average stroke volume. **a** Breakdown of the exercise frequency of the surveyed participants. **b** Comparison of the average stroke volume per exercise frequency (*n* = 31 participants). In the plot, each box spans from the first to the third quartile, with the horizontal line indicating the median. The whiskers extend up to 1.5 times the interquartile range beyond the box’s lower and upper bounds. **c** Distribution of beat-by-beat stroke volumes in the collected data for five minutes of the baseline state (pre-exercise) and five minutes after exercise.

Table 2 provides the demographics for the study population of 23 participants, consisting of 13 males and 10 females across a range of ages, weights and heights. Specifically, participants’ ages ranged from 21 to 58 years, and their body mass indexes (BMI) spanned from 19 to 42, covering the range of normal to obese. This variation highlights the high level of variability in demographics in the cohort. Additionally, participants with varying fitness levels were recruited to ensure a broad distribution of stroke volumes. The sample size was chosen based on relevant literature. The cohort of participants included individuals with a wide range of exercise habits, from those who rarely exercise to those who work out daily, as illustrated in Fig. 5a. The relationship between average stroke volume and exercise frequency for the participants is summarised in Fig. 5b. Generally, the median stroke volume increases with increasing exercise frequency, though there is a greater variation in stroke volumes among those exercising 3–4 times per week compared to other frequencies, possibly due to the types of exercise, where endurance exercises will have a different cardiac response than high-intensity exercises. Figure 5c provides the distribution of beat-by-beat stroke volume data collected in the study. A slight right shift is noticeable in the post-exercise data compared to pre-exercise, indicating a higher number of individual heartbeats with larger stroke volumes. On average, the mean stroke volume before exercise is 77 ± 13 ml, while after exercise, it increased slightly to 79 ± 14 ml. Comparing the average stroke volume per user before and after exercise, using a Wilcoxon signed-rank test, the difference is statistically significant with *p* < 0.05.

### Pre-processing

The frequency range of the heart signals captured inside the ear canal is less than 50 Hz^19^. As such, we downsample our collected audio to 600 Hz. We divide the audio into 10-s segments with an 8 s overlap between successive segments. We filter the signal with a 100 Hz low-pass filter to remove unrelated signal components. We compute a mel spectrogram^37^ (a representation of how a signal changes in frequency over time based on the mel scale, which mimics the way humans perceive sound) on our 10-se windows using 32 mel bins to accentuate low-frequency heart activity. We then interpolate this to 128 bins to match the size of the AudioMAE input in ref. ^37^. As illustrated in Fig. 4b, the output of this pipeline is a mel spectrogram of a 10-s window with size 128 × 1024.

### Deep learning architecture

Figure 4c, d provides an overview of the pipeline used in CardiAural. Motivated by the recent success of vision transformers in audio classification, which effectively capture intricate spatial and temporal patterns in spectrogram data using self-attention mechanisms^38,39^, we apply vision transformers to in-ear audio spectrograms. This approach enables the model to learn complex and robust features essential for accurate stroke volume estimation.

To achieve this, we use generative self-supervised learning implemented as a masking and reconstruction task on the input spectrogram. Self-supervised learning is a subset of unsupervised learning that uses self-generated challenges to create supervised tasks from unlabelled data^40^. With generative self-supervised learning, the challenge is to reconstruct the input sample, where the sample itself supervises the training of its reconstruction^41^. In our system, the challenge is spectrogram reconstruction whereby the model reconstructs portions of a spectrogram that have a binary mask applied to them using the unmasked portion as the label. Using self-supervised learning enables the model to learn meaningful and robust data representations from intricate in-ear heart signals, thus improving the model’s ability to generalise to new users and differing signal properties^40^.

Specifically, our pipeline uses the Audio-MAE model, which is an extension of a masked autoencoder from the image domain to audio^37^. At a high level, the network consists of a Transformer encoder and decoder. The network input is masked spectrograms (spectrograms with random binary patches applied to them) of audio and the network is only trained on the visible, unmasked patches. In doing so, the encoder extracts useful features from the masked spectrogram enabling the decoder to reconstruct the original spectrogram. The encoder and decoder use a stack of 12-layer ViT-Base Transformers^42^. However, the decoder enhances the basic transformer module to incorporate local attention so that the model takes into account the position and order of the spectrogram features, both of which are key to the semantics of the sound itself.

### Pre-training

Two additional key challenges in this work are the scarcity of labelled data and the need to enable the system to generalise to unseen subjects, thereby allowing new users to achieve accurate stroke volume estimations without the need for expensive labelled data collection. To address these challenges, we implement a two-stage training process that combines generative self-supervised pre-training with fine-tuning.

Specifically, the model is initialised using weights obtained by training on the AudioSet dataset^37,43^ AudioSet is an audio dataset containing ~2 million 10 s audio files from YouTube videos^43^. This is a very general audio dataset with 527 classes from music, to vehicle noises to a small subset of respiratory sounds, including snoring and breathing. While this dataset is useful for leveraging more general representations from audio, it is highly non-specific and the audio signals have vastly different properties to audio collected from in-ear microphones. For music data, the frequencies of interest range up to 16 kHZ. However, for in-ear audio data, the highest frequency of interest is 50 Hz^18,44^. Thus, for in-ear audio signals, we require more information to be extracted from lower frequency bands than from general audio datasets. To enable the model to learn generalisable features that are better related to our task, we pre-train the model using self-supervised learning with a dataset of unlabelled in-ear audio. The Earable dataset, as detailed in Table 3, contains in-ear audio data collected under stationary, or mostly stationary, scenarios including lying down, sitting, working at a desk, speaking etc. In total, we pre-trained our network with 34,940 10-s audio samples from the Earable dataset. As in ref. ^37^, we use unstructured masking for pretraining, where random pixels are removed, as shown in Fig. 4c. We use a masking ratio of 0.7, meaning that 70% of the pixels in the image are masked during pretraining. We trained the network for 300 epochs with a learning rate of 1*10^−3^ and a batch size of 64 using the mean square error (MSE) loss and used the final encoder in the fine-tuning step.

**Table 3:** Description of the generalised earable dataset used in the pre-training step

Dataset name	Description	Duration (minutes)
hEARt^18^	Heart rate estimation dataset encompassing 2 activities with 20 participants	89.7
RespEar^19,45^	Breathing rate estimation dataset encompassing 6 activities with 18 participants	632.3
In-ear cardiac sounds^46^	In-ear heart sounds segmentation dataset with different noise levels for 14 participants	89.5
Tidal volume sounds^47^	Tidal volume estimation dataset with stationary resting and cooldown (elevated heart and breathing rate) activities for 20 participants	353.2
Total		1164.7

### Fine-tuning

The process of fine-tuning is illustrated in Fig. 4d. When fine-tuning, we removed the decoder layers and retained only the encoder with a single fully connected layer on top for stroke volume predictions. We fine-tune this model with the smaller, highly specialised dataset specifically collected for stroke volume estimation. For fine-tuning, we use 2D masking as shown in Fig. 4d^37^ where we randomly mask bands of the spectrogram in frequency and in time with a masking ratio of 0.1. This enables the model to learn from local structures in the spectrogram while still using masking to force deeper learning of features. To address the dataset imbalance shown in Fig. 5c, we augment the dataset by duplicating and discarding samples to retain the median number of samples in the dataset per bin. This prevents the model from being biased towards more frequently occurring labels. We train the model using a learning rate of 1*10^−4^ and a batch size of 16 for 40 epochs.

We use a custom loss function which combines the MSE loss and a weighted MSE loss. The MSE loss, defined in Eq. (1), penalises errors across all target values equally, which helps the model to perform well on centrally located values. However, in this system, it is equally as important to accurately predict stroke volumes at the ranges of the distribution. To emphasise the importance of these points, we designed a weighted MSE loss which emphasises prediction errors towards the upper and lower range of the collected dataset. The loss is defined according to Eq. (2) where *y*_*i*_ are the ground truth values and ${\hat{y}}_{i}$ are the model predictions.

1 $${{{{\mathcal{L}}}}}_{MSE}=\frac{1}{n}\mathop{\sum }_{i=1}^{n}{({y}_{i}-{\hat{y}}_{i})}^{2}$$

2 $${{{{\mathcal{L}}}}}_{weighted}=\frac{1}{n}\mathop{\sum }_{i=1}^{n}{w}_{i}{({y}_{i}-{\hat{y}}_{i})}^{2}$$

In the weighted MSE loss, *w*_*i*_ is the weight assigned to each prediction, as defined in Eq. (3) and *μ* is the midpoint of the dataset. We normalise the weights by dividing them by the mean squared deviation of the lower or upper range, depending on whether *y*_*i*_ is less than or greater than *μ*. This normalisation ensures that the contributions from both the higher and lower target ranges remain balanced and prevent the weighted loss from disproportionately dominating the optimisation process.

3 $${w}_{i}=\left\{\begin{array}{ll}\frac{| {y}_{i}-\mu {| }^{2}}{mean(| {y}_{j}-\mu {| }^{2}\,for\,{y}_{j}\le \mu)},\quad &\,{\mbox{if}}\,\,{y}_{i}\le \mu \\ \frac{| {y}_{i}-\mu {| }^{2}}{mean(| {y}_{j}-\mu {| }^{2}\,for\,{y}_{j} > \mu)},\quad &{if}\,{y}_{i} > \mu \end{array}\right.$$

The overall loss function is a combination of the MSE loss and the weighted MSE loss, defined in Eq. (4), where *α* is set to 0.5 to balance the contributions of each loss component.

4 $${{{{\mathcal{L}}}}}_{custom}=\alpha {{{{\mathcal{L}}}}}_{weighted}+(1-\alpha){{{{\mathcal{L}}}}}_{MSE}$$

We fine-tune the model to predict the stroke volume per window and ultimately average together the predictions to compute the average stroke volume for a user. To evaluate model performance, we use leave-one-subject-out cross-validation. In this approach, the model is trained using data from all but one subject and tested on the data from the remaining subject. We train each model for a fixed number of epochs (20), and use the final model for testing. This method assesses the model’s ability to predict average stroke volume for a subject whose data it has not encountered before, mimicking the ideal use case for such a system.

### Evaluation metrics

We evaluate system performance using the mean absolute error (MAE) between the ground truth stroke volume and the calculated stroke volume, and the mean absolute percentage error (MAPE) between the two. We also assess performance using a Bland–Altman plot, which assesses the level of agreement of the measurement with the ground truth measurement. In addition, we evaluate the correlation between the two measurements using Pearson’s correlation analysis, and the level of agreement with the *R*^2^ score (the coefficient of determination). Finally, we assess whether the errors are acceptable by computing the percentage error (PE), which is defined as 1.96 times the standard deviation of the bias from the Bland–Altman plot, divided by the average ground truth stroke volume^6,22^. For a stroke volume measurement device to be accurate, it must have a PE of less than 30%^6,13,22^.

### Reporting summary

Further information on research design is available in the Nature Portfolio Reporting Summary linked to this article.

## Supplementary information

Supplementary Information

Reporting Summary

Transparent Peer Review file

## Source data

Source Data

## Data Availability

The in-ear audio data and the corresponding ground truth data generated by this study is sensitive as it can be identifiable. As such, an anonymised version of the in-ear data and the ground truth is made available exclusively for research purposes on reasonable request and can be obtained with an email to the corresponding author indicating the intended use: this to log and vet requests in terms of export control and purpose of the research. We aim to answer each request within two months. The source data for the figures is provided with the paper. Source data are provided with this paper.

## Code availability

The code underlying this study is not publicly available due to its proprietary nature but is available for non-commercial research and reproducibility purposes from the corresponding author upon request. Requests should include a description of the intended use and will be handled within two months.
