# Supplementary material for: Measuring cardiac stroke volume through in-ear audio sensing
Source: Nat Commun. 2026 Jul 17;17:8755. doi: 10.1038/s41467-026-75642-0 (PMC13494022; doi:10.1038/s41467-026-75642-0)
Supplement: Supplementary file 1 [file 41467_2026_75642_MOESM1_ESM.pdf]

Measuring Cardiac Stroke Volume Through In-ear Audio Sensing

This document provides high-level pseudocode corresponding to the methods described in the manuscript "Measuring Cardiac Stroke Volume Through In-ear Audio Sensing". It summarises the pre-processing pipeline, generative self-supervised pre-training procedure, and fine-tuning strategy used for stroke volume estimation from in-ear audio.

Algorithm 1 In-ear Audio Pre-processing

Require: Raw in-ear audio recording x
Ensure: Mel spectrogram segments $\{S_k\}$
Downsample x to 600 Hz
Apply 100 Hz low-pass filter
Segment x into 10 s windows with 8 s overlap
for each window x_k **do**
    Compute mel spectrogram with 32 mel bins
    Interpolate to 128 mel bins to obtain S_k
end for
return $\{S_k\}$

Algorithm 2 Generative Self-Supervised Pre-training

Require: Unlabelled in-ear audio dataset $\mathcal{D}_{\text{pre}}$
Ensure: Pre-trained encoder parameters θ_E
Initialise masked autoencoder with Transformer encoder and decoder
Initialise weights from AudioSet pre-training
for each spectrogram $S \in \mathcal{D}_{\text{pre}}$ **do**
    Apply random unstructured masking with ratio 0.7
    Encode visible patches with Transformer encoder
    Decode to reconstruct masked spectrogram patches
    $\mathcal{L}_{\text{rec}} \leftarrow \text{MSE}\Big(S_{\text{masked}}, \hat{S}_{\text{masked}}\Big)$
    Update encoder and decoder parameters to minimise $\mathcal{L}_{\text{rec}}$
end for
Discard decoder; retain encoder parameters θ_E
return θ_E

Algorithm 3 Fine-tuning with Leave-One-Subject-Out Cross-Validation

Require: Labelled dataset $\mathcal{D} = \{(S_i, y_i, p_i)\}$ with subject ID p_i
Require: Pre-trained encoder parameters θ_E
Ensure: Per-subject stroke volume estimates $\{\hat{y}_p\}$
Let $\mathcal{P}$ be the set of unique subjects in $\mathcal{D}$
for each subject $p \in \mathcal{P}$ **do**
    $\mathcal{D}_{\text{train}} \leftarrow \{(S_i, y_i) : p_i \neq p\}$
    $\mathcal{D}_{\text{test}} \leftarrow \{S_i : p_i = p\}$
    Initialise encoder parameters θ_E
    Initialise regression head parameters θ_h
    Define $\text{Encoder}(\cdot; \theta_E)$ and $\text{Head}(\cdot; \theta_h)$
    for each mini-batch $\{(S_i, y_i)\} \subset \mathcal{D}_{\text{train}}$ **do**
        Apply structured 2D masking in time and frequency (ratio 0.1)
        $z_i \leftarrow \text{Encoder}(S_i; \theta_E)$
        $\hat{y}_i \leftarrow \text{Head}(z_i; \theta_h)$
        $\mathcal{L}_{\text{MSE}} \leftarrow \frac{1}{n} \sum_i (y_i - \hat{y}_i)^2$
        $\mathcal{L}_{\text{weighted}} \leftarrow \frac{1}{n} \sum_i w_i (y_i - \hat{y}_i)^2$
        $\mathcal{L}_{\text{custom}} \leftarrow 0.5\, \mathcal{L}_{\text{weighted}} + 0.5\, \mathcal{L}_{\text{MSE}}$
        Update θ_E and θ_h to minimise $\mathcal{L}_{\text{custom}}$
    end for
    Predict window-level stroke volumes for $\mathcal{D}_{\text{test}}$ using (θ_E, θ_h)
    $\hat{y}_p \leftarrow$ mean of window-level predictions for subject p
end for
return $\{\hat{y}_p\}$
