# Supplementary material for: Measuring cardiac stroke volume through in-ear audio sensing
Source: Nat Commun. 2026 Jul 17;17:8755. doi: 10.1038/s41467-026-75642-0 (PMC13494022; doi:10.1038/s41467-026-75642-0)
Supplement: Supplementary file 2 [file 41467_2026_75642_MOESM2_ESM.pdf]

nature portfolio

Corresponding author(s): Kayla-Jade Butkow
Last updated by author(s): Sep 24, 2024

# Reporting Summary

Nature Portfolio wishes to improve the reproducibility of the work that we publish. This form provides structure for consistency and transparency in reporting. For further information on Nature Portfolio policies, see our Editorial Policies and the Editorial Policy Checklist.

## Statistics

For all statistical analyses, confirm that the following items are present in the figure legend, table legend, main text, or Methods section.

| n/a | Confirmed | |
|---|---|---|
| ☐ | ☒ | The exact sample size (*n*) for each experimental group/condition, given as a discrete number and unit of measurement |
| ☐ | ☒ | A statement on whether measurements were taken from distinct samples or whether the same sample was measured repeatedly |
| ☐ | ☒ | The statistical test(s) used AND whether they are one- or two-sided<br>*Only common tests should be described solely by name; describe more complex techniques in the Methods section.* |
| ☒ | ☐ | A description of all covariates tested |
| ☒ | ☐ | A description of any assumptions or corrections, such as tests of normality and adjustment for multiple comparisons |
| ☐ | ☒ | A full description of the statistical parameters including central tendency (e.g. means) or other basic estimates (e.g. regression coefficient) AND variation (e.g. standard deviation) or associated estimates of uncertainty (e.g. confidence intervals) |
| ☐ | ☒ | For null hypothesis testing, the test statistic (e.g. *F*, *t*, *r*) with confidence intervals, effect sizes, degrees of freedom and *P* value noted<br>*Give P values as exact values whenever suitable.* |
| ☒ | ☐ | For Bayesian analysis, information on the choice of priors and Markov chain Monte Carlo settings |
| ☒ | ☐ | For hierarchical and complex designs, identification of the appropriate level for tests and full reporting of outcomes |
| ☐ | ☒ | Estimates of effect sizes (e.g. Cohen's *d*, Pearson's *r*), indicating how they were calculated |

*Our web collection on statistics for biologists contains articles on many of the points above.*

## Software and code

Policy information about availability of computer code

| | |
|---|---|
| Data collection | We used a python script to sample audio from our earbud device. This uses python 3.7 on a Raspberry Pi 4 and saves raw audio data onto the Raspberry Pi. Data was subsequently saved on a secure server in the Department of Computer Science, University of Cambridge. |
| Data analysis | The framework was implemented in Python 3.12 and Pytorch 2.3.0, based on AudioMAE (https://github.com/facebookresearch/AudioMAE/tree/main). |

For manuscripts utilizing custom algorithms or software that are central to the research but not yet described in published literature, software must be made available to editors and reviewers. We strongly encourage code deposition in a community repository (e.g. GitHub). See the Nature Portfolio guidelines for submitting code & software for further information.

## Data

Policy information about availability of data

All manuscripts must include a data availability statement. This statement should provide the following information, where applicable:

- Accession codes, unique identifiers, or web links for publicly available datasets
- A description of any restrictions on data availability
- For clinical datasets or third party data, please ensure that the statement adheres to our policy

The in-ear audio data and the corresponding ground truth data generated by this study is sensitive as it can be identifiable. As such, an anonymised version of the in-ear data is made available exclusively for research purposes on reasonable request and can be obtained with an email request to strokevolumeinear@gmail.com.

## Research involving human participants, their data, or biological material

Policy information about studies with human participants or human data. See also policy information about sex, gender (identity/presentation), and sexual orientation and race, ethnicity and racism.

| | |
|---|---|
| Reporting on sex and gender | We have reported errors based on sex for the cohort. Sex was collected based on self-reporting. In total, the dataset contains data collected from 13 males and 10 females. |
| Reporting on race, ethnicity, or other socially relevant groupings | We have not reported on race, ethnicity or other groupings in our manuscript. |
| Population characteristics | 23 healthy volunteers aged 21 to 58, with weights from 55kg to 113kg and heights from 158cm to 194cm. Stroke volume measurements ranged from 45ml to 118ml. |
| Recruitment | Participants were recruited from the Department of Computer Science at the University of Cambridge from friends of the authors and coworkers and staff in the department. Participation was voluntary and could be withdrawn at any time. |
| Ethics oversight | The study was approved by the ethics committee of the Department of Computer Science at the University of Cambridge, with ID #2238. Participants signed an informed consent form prior to starting the data collection. |

Note that full information on the approval of the study protocol must also be provided in the manuscript.

# Field-specific reporting

Please select the one below that is the best fit for your research. If you are not sure, read the appropriate sections before making your selection.

☒ Life sciences ☐ Behavioural & social sciences ☐ Ecological, evolutionary & environmental sciences

For a reference copy of the document with all sections, see nature.com/documents/nr-reporting-summary-flat.pdf

# Life sciences study design

All studies must disclose on these points even when the disclosure is negative.

| | |
|---|---|
| Sample size | Sample size was chosen based on relevant literature. The dataset consists of 23 healthy participants. |
| Data exclusions | No data was excluded from analysis. |
| Replication | Datasets are available from the authors upon reasonable request. Initialisation weights can be obtained from https://github.com/facebookresearch/AudioMAE/tree/main. The code for this study and the models generated are proprietary or confidential in nature and therefore cannot be shared. |
| Randomization | No randomisation was performed during participant recruitment. Participants were recruited without any age, gender or racial bias. |
| Blinding | Anonymous IDs were assigned to all study participants to protect their privacy. No binding was conducted as it does not impact the performance comparison between predicted stroke volumes from in-ear audio and the clinical reference device |

# Reporting for specific materials, systems and methods

We require information from authors about some types of materials, experimental systems and methods used in many studies. Here, indicate whether each material, system or method listed is relevant to your study. If you are not sure if a list item applies to your research, read the appropriate section before selecting a response.

## Materials & experimental systems

| n/a | Involved in the study | |
|---|---|---|
| ☒ | ☐ | Antibodies |
| ☒ | ☐ | Eukaryotic cell lines |
| ☒ | ☐ | Palaeontology and archaeology |
| ☒ | ☐ | Animals and other organisms |
| ☒ | ☐ | Clinical data |
| ☒ | ☐ | Dual use research of concern |
| ☒ | ☐ | Plants |

## Methods

| n/a | Involved in the study | |
|---|---|---|
| ☒ | ☐ | ChIP-seq |
| ☒ | ☐ | Flow cometry |
| ☒ | ☐ | MRI-based neuroimaging |

## Plants

| | |
|---|---|
| Seed stocks | *Report on the source of all seed stocks or other plant material used. If applicable, state the seed stock centre and catalogue number. If plant specimens were collected from the field, describe the collection location, date and sampling procedures.* |
| Novel plant genotypes | *Describe the methods by which all novel plant genotypes were produced. This includes those generated by transgenic approaches, gene editing, chemical/radiation-based mutagenesis and hybridization. For transgenic lines, describe the transformation method, the number of independent lines analyzed and the generation upon which experiments were performed. For gene-edited lines, describe the editor used, the endogenous sequence targeted for editing, the targeting guide RNA sequence (if applicable) and how the editor was applied.* |
| Authentication | *Describe any authentication procedures for each seed stock used or novel genotype generated. Describe any experiments used to assess the effect of a mutation and, where applicable, how potential secondary effects (e.g. second site T-DNA insertions, mosiacism, off-target gene editing) were examined.* |
