# Supplementary material for: Measuring cardiac stroke volume through in-ear audio sensing
Source: Nat Commun. 2026 Jul 17;17:8755. doi: 10.1038/s41467-026-75642-0 (PMC13494022; doi:10.1038/s41467-026-75642-0)
Supplement: Supplementary file 3 [file 41467_2026_75642_MOESM3_ESM.pdf]

nature portfolio

Peer Review File

Measuring Cardiac Stroke Volume Through In-ear Audio Sensing

Corresponding Author: Professor Cecilia Mascolo

This file contains all reviewer reports in order by version, followed by all author rebuttals in order by version.

A version of this paper was originally rejected for publication by Nature Communications, however that decision was reconsidered after appeal by the authors.

Version 1:

Reviewer comments:

Reviewer #1

(Remarks to the Author)
Referee Report for the Manuscript "Measuring Cardiac Stroke Volume Through In-ear Audio Sensing"

General Comments: The manuscript presents an approach to estimate stroke volume using in-ear audio sensing, a method which could enhance non-invasive cardiovascular monitoring. The description of the machine learning method used for stroke volume estimation is notably clear and comprehensive, providing a good understanding of the process.

Specific Comments:

1. Data Utilization and Clarification: The manuscript would benefit from a more detailed explanation of the data used for training and validating the model. Specifically, it should include information on how much data was collected per patient.

2. Figure 1 Suggestion: Figure 1 shows stroke volume changes which appear to follow respiratory sinus arrhythmia. It would be informative to overplot IBI (Inter-Beat Interval) intervals from ECG to examine the correlation between IBI and stroke volume more explicitly. This addition could provide deeper insights into the physiological interactions captured by the system.

3. Bland-Altman Analysis: While the Bland-Altman analysis is well-executed, presenting it per patient as well as per activity could offer a clearer view of the variability and accuracy across individual subjects. This could help in understanding patient-specific performance of the algorithms.

4. Discussion of Limitations: The manuscript commendably presents a feasibility study. However, the limitations section could be expanded to address the small sample size, which may lead to underestimation of the errors. This concern is particularly pertinent since Table 1 indicates that the Earable has an 11.05% error, significantly lower than other methods reported in the literature, which have error rates ranging from 16% to 37%. The authors also show that using only demographic variables can yield a 15% error. Discussing how the sample size and participant characteristics might have influenced these results would provide a more balanced view and enhance scientific rigor. Additionally, it would be beneficial to mention the error associated with the gold standard used as the stroke volume reference in the study.

5. Technical Specifications of the Microphone Response: The manuscript briefly mentions the response of the microphone used in the earables, but lacks description concerning the frequency range utilized in the analysis and whether any additional filters were applied. Clarifying these technical details would greatly enhance the understanding of how the audio data was processed and analyzed, ensuring the integrity and reproducibility of the results. It is crucial to specify the frequency response of the microphone, particularly whether it aligns with the key frequencies of interest for capturing cardiovascular sounds, which can go significantly below 20 Hz.

Conclusion: The study is a commendable effort in the field of biomedical engineering, demonstrating a method to measure stroke volume non-invasively. Addressing the above points would enhance the manuscript's clarity.

(Remarks on code availability)

Reviewer #3

(Remarks to the Author)
Overall, this paper presents an interesting and novel contribution to wearable physiological sensing by demonstrating that stroke level can be accurately measured on the ear. There are a few issues in the paper that can be addressed in revision but I am excited to see this in publication.

-- What are the noteworthy results?

This paper demonstrates that it is feasible to detect stroke level using in-ear microphones and ear-based devices using deep learning approaches to what appears to be a very good level of accuracy. This nicely adds to the growing work on using wearable devices for physiological measurement, especially relating to cardiac output.

-- Will the work be of significance to the field and related fields? How does it compare to the established literature? If the work is not original, please provide relevant references.

This builds on the principle of using the occlusion effect by sealing the ear canal and using internal facing microphones, which has been shown to be effective for other physiological signals (e.g., https://doi.org/10.1016/j.pmcj.2024.101913). However, I am not aware of any work that attempts to predict stroke level. Using wearables for cardiac measurements is an emerging and growing area with a lot of research interest (e.g. https://doi.org/10.3389/fphys.2020.568886, https://www.tandfonline.com/doi/full/10.1080/17434440.2021.2014321, or https://dl.acm.org/doi/abs/10.1145/3550314).

These results are significant because they pave the way for continual measurement of stroke level in a practical form factor. However, I think the paper could better motivate and frame the significance of this. For example, the wearable devices paragraph in the motivation seems to be targeting a different set of use cases to the medical grade instruments. Is this earable device being contrasted against both or more so the wearables? If the latter, what are the use cases for these - are they still in clinic usage? On this note, it would be good to see some motivation for the need for continuous stroke volume monitoring after the medical and before the wearable section in the introduction.

-- Does the work support the conclusions and claims, or is additional evidence needed?

The abstract and introductions seem to be a bit misleading because it poses how "no solution relies solely on the sensors of commodity wearable devices". While I appreciate the proposed solution relies on sensors that are in commodity wearable devices, the device studied in this paper is not an unmodified commodity wearable devices, and instead uses a sealed ear canal which is something I have not seen on commodity earphones. This research is a very positive step, however because the fundamental sensing principle appears to be dependent upon the ear canal being sealed (line 382) it is not clear how practical this is for everyday use, and whether or not it would introduce other potential issues (e.g., increased risk of ear infection). In revision, the framing of these claims should make it clearer to the reader that this is a step towards, but not realisation of stroke level measurement on commodity wearables, and discuss the limitations around the requirement to seal the ear canal.

In terms of the generalisability of the approach, this paper demonstrates the approach works well for healthy participants in an exercise protocol. This is commendable and very interesting. The paper highlights the need for more validation across a wider range of stroke volumes and how the current approach is more error prone at the extreme ends which warrants future research. In light of the low standard deviation and difficulty in estimating stroke volume at the extreme ends, it would be good to see a more in-depth discussion of activities at the stroke levels for which this technology has been shown to be effective.

One element of concern is the ground truth used for the stroke level is the Finapres Nova, a non-invasive method for measuring stroke level. This does not appear to be the gold standard for measuring stroke volume (e.g. see https://pmc.ncbi.nlm.nih.gov/articles/PMC9075258/). I think the paper needs to include more information about how accurate the Finapres Nova is relative to the gold standard of stroke level measurement, because these results are relative. The claims that this is below the 30% deemed acceptable is a bit harder to judge because in the Critchley and Critchley paper the methods are compared against thermodilution, dye dilution, or the Fick method. This needs to be better highlighted in the paper and reflected in limitations.

Building upon this, Table 1 appears to be misleading because the ground truths vary between research papers and therefore it is not a like for like comparison and is potentially misleading. Ganti et al compare to cardiovascular magnetic resonance imaging. Dvir et al. use pulse contour cardiac output, Wang et all use Physio Flow PF-05, Yazdi et al use scale and direct Fick measurements of stroke volume and cardiac output before and immediately after invasive cardiopulmonary

exercise test. These should be more accurately reflected in the table, and commentary provided about the different measures used for ground truths.

-- Are there any flaws in the data analysis, interpretation and conclusions? Do these prohibit publication or require revision?
-- Is the methodology sound? Does the work meet the expected standards in your field?

The paper claims that is "evident" that changes in the in-ear audio correspond to changes in the stroke volume signal in figure 1 (line 173). As a reader, this isn't evident and I think it can be made clearer in the figure why exactly this is evident. IN particular, the changes described in lines 176 -178 could be from a variety of different physiological effects including increased heart rate, respiration rate etc. What are the exact reasons these are supposed to link with stroke volume? These could be made clearer to the reader in text and in the figure.

Lines 195-197 claims that the weak correlations prove that handcrafted features are not the optimal way to achieve good results. However, I disagree. It shows the individual components do not achieve good results - but what about non deep-learning methods like mixed effect linear regression models that could take these features and potentially predict stroke rate based on multiple dimensions, and in a more interpretable way? I note later in the paper there is a discussion about using SVM for classification which is a really nice inclusion, and better motivates the use of deep learning. However, I think the language needs to be tone down a bit more with respect to what is underpinning some of the justifications.

In 2.2 line 216, it would be good to see the 95% limits of agreement reported here.

The paper uses leave one out cross validation - however, it would be good to see what the variance is for the cross-validation.

-- Is there enough detail provided in the methods for the work to be reproduced?

The paper provides a good level of details for replication, however the specific details about the custom ear device are missing. For replicability, it would be good to discuss what exact components were used (e.g., by labelling figure 3).

(Remarks on code availability)
No code or data provided for review.

Version 2:

Reviewer comments:

Reviewer #1

(Remarks to the Author)
My comments have been incorporated into the revised version, and I recommend the manuscript for publication.

(Remarks on code availability)

Reviewer #3

(Remarks to the Author)
I would like to sincerely thank the authors for their thoughtful and thorough engagement with the reviewer comments. It is clear that considerable care was taken in addressing the feedback, and I appreciate the effort put into both the detailed responses and the revisions to the manuscript. The changes made have not only clarified several key points but have also strengthened the overall quality and coherence of the paper. In my view, these revisions have led to a significant improvement in the work. I am now fully satisfied with the current version and, as such, I am pleased to recommend the paper for acceptance.

(Remarks on code availability)

Open Access This Peer Review File is licensed under a Creative Commons Attribution 4.0 International License, which permits use, sharing, adaptation, distribution and reproduction in any medium or format, as long as you give appropriate credit to the original author(s) and the source, provide a link to the Creative Commons license, and indicate if changes were made.
In cases where reviewers are anonymous, credit should be given to 'Anonymous Referee' and the source.
The images or other third party material in this Peer Review File are included in the article's Creative Commons license, unless indicated otherwise in a credit line to the material. If material is not included in the article's Creative Commons license and your intended use is not permitted by statutory regulation or exceeds the permitted use, you will need to obtain permission directly from the copyright holder.
To view a copy of this license, visit https://creativecommons.org/licenses/by/4.0/

Dear Reviewers,

Thank you for your thorough comments on our paper. We have tried to answer and address them to the best of our ability.

Reviewer #1

Comment*: The manuscript presents an approach to estimate stroke volume using in-ear audio sensing, a method which could enhance non-invasive cardiovascular monitoring. The description of the machine learning method used for stroke volume estimation is notably clear and comprehensive, providing a good understanding of the process.*

Response: Thanks for the comment, and we are pleased that the presentation was deemed clear and comprehensive.

Comment*: Data Utilization and Clarification: The manuscript would benefit from a more detailed explanation of the data used for training and validating the model. Specifically, it should include information on how much data was collected per patient.*

Response: In Section 4.7, we described the data used for pre-training, which includes the earable data described in Table 2. In section 4.8, we state that “We fine-tune this model with the smaller, highly specialised dataset specifically collected for stroke volume estimation.”. We have added the following to Section 4.8 to clarify that we did not use any data to validate the linear probe, but rather trained for a fixed number of epochs.

> We train each model for a fixed number of epochs (20), and use the final model for testing.

We have also added the following paragraph to Section 4.3 to emphasise how much data we collected per participant:

> In total, we collected 16 minutes of data per participant, and we analyse the data collected in the resting state and the recovery state.

Comment*:* Figure 1 Suggestion: Figure 1 shows stroke volume changes which appear to follow respiratory sinus arrhythmia. It would be informative to overplot IBI (Inter-Beat Interval) intervals from ECG to examine the correlation between IBI and stroke volume more explicitly. This addition could provide deeper insights into the physiological interactions captured by the system.

Response: We have modified Figure 1, adding IBI from ECG and updating the caption accordingly.

Comment: *Bland-Altman Analysis: While the Bland-Altman analysis is well-executed, presenting it per patient as well as per activity could offer a clearer view of the variability and accuracy across individual subjects. This could help in understanding patient-specific performance of the algorithms.*

Response: We added a subfigure to Figure 2 (Figure 2c) which presents a Bland Altman plot of the overall average stroke volume per participant to explain the participant-specific performance. We also added the following text to Section 2.2.

> Figure 2 (c) provides a Bland-Altman analysis of the overall average stroke volume per participant. From this plot, it is clear that one participant is an outlier in the system, achieving an error that exceeds the limits of agreement of the Bland-Altman plot. However, generally, the agreement between the measurements and the ground truth is good.

Comment: *Discussion of Limitations: The manuscript commendably presents a feasibility study. However, the limitations section could be expanded to address the small sample size, which may lead to underestimation of the errors. This concern is particularly pertinent since Table 1 indicates that the Earable has an 11.05% error, significantly lower than other methods reported in the literature, which have error rates ranging from 16% to 37%. The authors also show that using only demographic variables can yield a 15% error. Discussing how the sample size and participant characteristics might have influenced these results would provide a more balanced view and enhance scientific rigor.*

Response: Thank you for this comment. We agree that a deeper discussion on the limitations of our cohort is necessary. We have added the following sentence to Section 3:

> This paper presents a feasibility study into a new modality of stroke volume estimation, and as such, the study cohort includes a limited number of individuals. Although our participant pool has a similar size to the other studies cited in this work, we acknowledge that the size of the cohort might have influenced the results, because of the limited variance. Thus, a fair comparison between our work and other studies from the literature is difficult. We also highlight that we have conducted the study with a cohort of healthy individuals: we expect that the variance would be higher in a mixed cohort (control and patients), which might impact the results.

Comment: *It would be beneficial to mention the error associated with the gold standard used as the stroke volume reference in the study.*

Response: We have added the following sentence to Section 4.3 to mention the error:

> Once calibrated, when participants are lying down, Finapres NOVA has a mean bias of 0%+-4.2% compared to echocardiogram [33]. There is thus good agreement between the two devices, meaning that our results experience minimal bias compared to ground truth readings taken from echocardiogram.

Comment: *Technical Specifications of the Microphone Response: The manuscript briefly mentions the response of the microphone used in the earables, but lacks description concerning the frequency range utilized in the analysis and whether any additional filters were applied. Clarifying these technical details would greatly enhance the understanding of how the audio data was processed and analyzed, ensuring the integrity and reproducibility of the results. It is crucial to specify the frequency response of the microphone, particularly whether it aligns with the key frequencies of interest for capturing cardiovascular sounds, which can go significantly below 20 Hz.*

Response: Section 4.5 describes our preprocessing steps on the audio. In this section, we state that we apply a 100Hz low-pass filter to the downsampled audio. We do not apply any other filtering to the audio. We have added the name of the microphone (Knowles SPU1410LR5H-QB) to the paragraph for better reproducibility. We have also adapted the following text for clarity:

> Each earbud contains a small analogue microphone (Knowles SPU1410LR5H-QB) with a flat frequency response at low frequencies (10-100Hz), enabling the capture of low-frequency in-ear heart sounds between 10 and 100Hz.

Comment: *The study is a commendable effort in the field of biomedical engineering, demonstrating a method to measure stroke volume non-invasively. Addressing the above points would enhance the manuscript's clarity.*

Response: Thanks for the positive comments, which have helped us improve the quality of our manuscript.

Reviewer #3

Comment: *Overall, this paper presents an interesting and novel contribution to wearable physiological sensing by demonstrating that stroke level can be accurately measured on the ear. There are a few issues in the paper that can be addressed in revision but I am excited to see this in publication.*

Response: Thank you for your positive comments and feedback, which we have tried to answer to the best of our abilities.

Comment:
-- What are the noteworthy results?

This paper demonstrates that it is feasible to detect stroke level using in-ear microphones and ear-based devices using deep learning approaches to what appears to be a very good level of accuracy. This nicely adds to the growing work on using wearable devices for physiological measurement, especially relating to cardiac output.

-- Will the work be of significance to the field and related fields? How does it compare to the established literature? If the work is not original, please provide relevant references.

This builds on the principle of using the occlusion effect by sealing the ear canal and using internal facing microphones, which has been shown to be effective for other physiological signals (e.g., https://doi.org/10.1016/j.pmcj.2024.101913). However, I am not aware of any work that attempts to predict stroke level. Using wearables for cardiac measurements is an emerging and growing area with a lot of research interest (e.g. https://doi.org/10.3389/fphys.2020.568886, https://www.tandfonline.com/doi/full/10.1080/17434440.2021.2014321, or https://dl.acm.org/doi/abs/10.1145/3550314).

These results are significant because they pave the way for continual measurement of stroke level in a practical form factor.

Response: We are grateful for these positive comments and for having highlighted the aims and methodology of the work.

Comment: *I think the paper could better motivate and frame the significance of this. For example, the wearable devices paragraph in the motivation seems to be targeting a different set of use cases to the medical grade instruments. Is this earable device being contrasted against both or more so the wearables? If the latter, what are the use cases for these - are they still in clinic usage? On this note, it would be good to see some motivation for the need for continuous stroke volume monitoring after the medical and before the wearable section in the introduction.*

Response: We agree with the reviewer and at the start of the wearables section, we have edited the first sentence to emphasise that we are comparing to wearables rather than medical devices and to indicate possible use cases of continuous monitoring of stroke volume:

> Wearable devices have emerged as an alternative for non-invasive stroke volume estimation outside of clinical settings. By enabling continuous monitoring of cardiovascular health, they can help detect deviations over time and potentially support early identification of cardiovascular disease. Around 2% of the adult population are estimated to have undiagnosed heart failure [11], with misdiagnosis rates in general practice reaching up to 68% [12]. Continuous stroke volume assessment through wearables therefore offers a novel opportunity to detect early signs of pathophysiology, enabling earlier diagnosis and intervention. Beyond clinical use, these devices also provide value in tracking cardiovascular fitness across broader populations.

Comment: *The abstract and introductions seem to be a bit misleading because it poses how "no solution relies solely on the sensors of commodity wearable devices". While I appreciate the proposed solution relies on sensors that are in commodity wearable devices, the device studied in this paper is not an unmodified commodity wearable devices, and instead uses a sealed ear canal which is something I have not seen on commodity earphones. This research is a very positive step, however because the fundamental sensing principle appears to be dependent upon the ear canal being sealed (line 382) it is not clear how practical this is for everyday use, and whether or not it would introduce other potential issues (e.g., increased risk of ear infection). In revision, the framing of these claims should make it clearer to the reader that this is a step towards, but not realisation of stroke level measurement on commodity wearables, and discuss the limitations around the requirement to seal the ear canal.*

Response: Many currently commercial earbuds have active noise cancellation which includes a need to seal the ear canal to achieve high-quality noise cancellation. This includes the Airpods Pro 2, Pixel Buds Pro, Galaxy Buds Pro 2. The only reason we could not apply our algorithms directly to these devices is that these providers do not yet expose the APIs of the in-ear microphone. However, our algorithms are applicable to any of these commercial devices once APIs are created to access this sensor. We have explained this in

the manuscript in the paragraphs at lines 100 and 400. We have however followed the advice of the reviewer and added an additional sentence at the end of Section 4.2 to clarify this further:

> We note that while we had to develop our own hardware prototype to prove our concepts, our algorithms are independent of this hardware and would be able to run on any commercial earbuds providing noise-cancelling functionality. To achieve the seal necessary for good performance, the user will be required to undergo an earbud fit test (as is implemented on the Apple AirPods Pro) to pick the eartips that fit their ears best.

Comment: *In terms of the generalisability of the approach, this paper demonstrates the approach works well for healthy participants in an exercise protocol. This is commendable and very interesting. The paper highlights the need for more validation across a wider range of stroke volumes and how the current approach is more error prone at the extreme ends which warrants future research. In light of the low standard deviation and difficulty in estimating stroke volume at the extreme ends, it would be good to see a more in-depth discussion of activities at the stroke levels for which this technology has been shown to be effective.*

Response: We have added text to our Discussion Section which highlights how future tests will include assessing stroke volume changes under running and cycling activities, as these are known to have an effect on stroke volume. This will allow us to have a more robust range of values and investigate the effect of these activities.

> Our current experimental protocol includes an isometric squeezing test (Section 2.3), and we demonstrate the change in stroke volume between pre and post exercise. Our future tests will include comparative analysis with other activities to widen the range of stroke volumes and the variability in the dataset. In particular, we aim to compare the effect of cycling and running as this has been studied in the literature [26].

Comment: *One element of concern is the ground truth used for the stroke level is the Finapres Nova, a non-invasive method for measuring stroke level. This does not appear to be the gold standard for measuring stroke volume (e.g. see https://pmc.ncbi.nlm.nih.gov/articles/PMC9075258/). I think the paper needs to include more information about how accurate the Finapres Nova is relative to the gold standard of stroke level measurement, because these results are relative. The claims that this is below the 30% deemed acceptable is a bit harder to judge because in the Critchley and Critchley paper the methods are compared against thermodilution, dye dilution, or the Fick method. This needs to be better highlighted in the paper and reflected in limitations.*

Response: We have added the following text to Section 4.3 to explain the use of the Finapres over the Direct Fick method:

> The Finapres NOVA was selected for a number of reasons: Firstly, although the gold standard for measuring stroke volume is using thermodilution (direct Fick method), this involves an invasive catheter placed in the pulmonary artery, and is mainly used in critical care settings. Since we conducted this study on a healthy population, it was not ethically advisable to use this technique. Secondly, although cardiac magnetic resonance imaging could be employed to measure stroke volume, there are a

number of contraindications (presence of metal devices, renal dysfunction, contrast allergy, claustrophobia) and the utility of performing exercise whilst in an MRI machine is very limited. Additionally, concurrent use of a metallic earbud is contraindicated within an MRI. Therefore, we employed the use of a non-invasive device such as Finapres since it is one of only a few devices used in clinical research to assess beat-to-beat stroke volume.

However, in order to have an equivalent gold standard device for comparison for our study, we used non-invasive 2D echocardiography. This has previously been compared to invasive thermodilution in a meta-analysis [34] of the two methods. This study reported a median bias of –0.12 L/min with limits of agreement ±0.94 L/min, and a median correlation coefficient of 0.827 (range: 0.140–0.998), indicating strong agreement between the two methods. The paper concluded that the two were interchangeable. Similarly, a study in cardiac patients [35] found a high correlation (r = 0.91) and an average stroke volume difference of approximately 5 mL between echocardiography and the Fick method, further validating echocardiography as a reliable reference for calibration.

The FDA approval of Finapres mentions that the device should be calibrated to ensure accuracy. As such, we used echocardiogram as the best non-invasive gold standard calibration method for the Finapres in our outpatient, healthy volunteer study. Stroke volume was calculated using the GE Vivid IQ ultrasound system as the product of the left ventricular outflow tract (LVOT) cross-sectional area and the Doppler flow envelope recorded at the LVOT [7]. The average of three cardiac cycles was used. Once calibrated, when participants are lying down, Finapres NOVA has a mean bias of 0%+-4.2% compared to echocardiogram [36].

To set up the Finapres, a medical-grade three-lead ECG electrode was connected to each participant's chest, and a blood pressure cuff was placed on their non-dominant arm for calibration. The system was calibrated using each participant's stroke volume measurements from an echocardiogram. The Finapres was also calibrated using resting blood pressure measurements from the arm, taken while participants were lying down.

We have also added the following text to the discussion in Section 3:
Additionally, we acknowledge that while the Finapres NOVA is FDA approved for stroke volume monitoring, it is not the gold-standard clinical method for such measurements. However, we believe that its use is appropriate to validate our findings on a healthy population where invasive catheters as used in the gold-standard technique are inappropriate.

Comment: *Building upon this, Table 1 appears to be misleading because the ground truths vary between research papers and therefore it is not a like for like comparison and is potentially misleading. Ganti et al compare to cardiovascular magnetic resonance imaging. Dvir et al. use pulse contour cardiac output, Wang et all use Physio Flow PF-05, Yazdi et al use scale and direct Fick measurements of stroke volume and cardiac output before and immediately after invasive cardiopulmonary exercise test. These should be more accurately*

reflected in the table, and commentary provided about the different measures used for ground truths.

Response: We have added information about the ground truth devices used in Table 1. We have also added a sentence to the discussion on Table 1:

> However, it is important to note that all previous studies used different ground truth devices for comparison, implying that there is potential for bias in the results due to inherent bias in the ground truth measurement device. Specifically, the Direct Fick method used by Yadzi et al. [6] is the clinical gold-standard for stroke volume estimation, and the standard for which Critchley and Critchley's [24] 30% PE is devised. Thus, although our results look promising and the Finapres has been shown to have a high agreement with echocardiogram-derived stroke volume, further work is required to compare our estimations to measurements from the Direct Fick method to assess clinical suitability.

Comment: *The paper claims that is "evident" that changes in the in-ear audio correspond to changes in the stroke volume signal in figure 1 (line 173). As a reader, this isn't evident and I think it can be made clearer in the figure why exactly this is evident. IN particular, the changes described in lines 176 -178 could be from a variety of different physiological effects including increased heart rate, respiration rate etc. What are the exact reasons these are supposed to link with stroke volume? These could be made clearer to the reader in text and in the figure.*

Response: We agree that our initial phrasing was too strong regarding the observations in this image. We have now adjusted the image to include the interbeat intervals and adjusted the text as follows:

> We present an example of ground truth stroke volume collected from two participants and their corresponding in-ear audio in Figure 1. The corresponding ECG signals and their interbeat intervals (IBIs) are also collected to validate the effectiveness of in-ear audio for capturing cardiac activity. Figure 1 (a,b) present the stroke volume for one participant before and after isometric exercise respectively, and (c,d) present the same for a second participant. For each participant, 60 seconds of data per condition is shown. For Participant 1, the mean stroke volume in the 60 seconds was 91mL before exercise and 96mL afterwards, with an average heart rate of 70BPM. For Participant 2, the mean stroke volumes were 60mL and 65mL, respectively, with a heart rate of 61BPM. The two participants are the same age, with different fitness levels (as deduced from their exercise frequency) which is reflected in the different stroke volumes. Moreover, the alignment of the peaks of the in-ear heart signals and the ECG shows the correlation between the two signals. This provides evidence that the variations in the in-ear signals are due to heart activity.
>
> Immediately following isometric exercise, both participants exhibit an increase in the amplitude of the in-ear audio signal (Figure 1 (b,d)). This increase corresponds with elevated stroke volumes, which is consistent with expected physiological response to exercise. As the participants return to rest, the stroke volume gradually decreases, and this trend is mirrored by a reduction in the in-ear audio amplitude and a return of the IBI to baseline, indicating a lowering of heart rate and cardiac demand. In

contrast, during the resting condition (Figure 1 (a,c)), the stroke volume remains relatively stable, and the in-ear waveform shows minimal variation over time.

This example illustrates that the in-ear signal reflects changes in stroke volume, not just due to heart rate but also due to beat-to-beat variability in cardiac output, as shown in the IBI and SV signals.

Comment: *Lines 195-197 claims that the weak correlations prove that handcrafted features are not the optimal way to achieve good results. However, I disagree. It shows the individual components do not achieve good results - but what about non deep-learning methods like mixed effect linear regression models that could take these features and potentially predict stroke rate based on multiple dimensions, and in a more interpretable way? I note later in the paper there is a discussion about using SVM for classification which is a really nice inclusion, and better motivates the use of deep learning. However, I think the language needs to be tone down a bit more with respect to what is underpinning some of the justifications.*

Response: We agree with the reviewer and have now toned down those lines. The sentence now reads as:

This shows that in-ear audio has the potential to estimate stroke volume, however, handcrafted features alone struggle to generalise to differing signal properties. In Section 2.4, we provide some evidence of this limited generalisability to contextualise the predictive power of these features. Deep learning models have an inherent ability to approximate complex relationships and identify intricate patterns and features from data, making them a natural choice to solve this challenge.

Comment: *In 2.2 line 216, it would be good to see the 95% limits of agreement reported here.*

Response: We have added these results as follows:

Overall, our system can predict stroke volume from in-ear audio signals for unseen subjects with a mean absolute error (MAE) of 5.24ml, a mean absolute percentage error (MAPE) of 6.83% and 95% limits of agreement of -9.35ml to 9.31ml when compared to the clinical standard Finapres Nova.

Comment: *The paper uses leave-one-out cross-validation - however, it would be good to see what the variance is for the cross-validation.*

Response: We have added the following text to section 2.2:

When testing the system using a 70-30 cross validation, whereby 70% of all users data is used for training and the remaining 30% of the data is used for testing, we are able to achieve an error of 2.6ml+-1.88ml (3.57%) in estimating the overall average stroke volume for a participant. This shows that there is very low error and variance when seen data is used for model training.

Comment: *The paper provides a good level of details for replication, however the specific details about the custom ear device are missing.*

For replicability, it would be good to discuss what exact components were used (e.g., by labelling figure 3).

Response: Our main innovation is in the algorithms, and as mentioned above, our algorithms are applicable to any active noise cancelling earbuds once access to the microphone stream can be obtained. We have, however, also now added more details to the caption of Figure 3. In particular, the caption now reads:

> Fig 3: Exploded schematic of the custom earable system for in-ear audio collection. A) Custom 3D printed earhook based on the design of Roddiger et al. [45]. B) speaker (a standard true wireless stereo 8mm, 16 Ohm earbud speaker) c) Knowles SPU1410LR5H-QB microphone d) 3D printed front cap e) rubber ear tip f) wire g) custom PCB containing one MCP6004 non-inverting operational amplifier per channel (earbud). These amplifiers are controlled by potentiometers, allowing for adjustable gain if required. The gain value was set to unity for all data collection exercises. The PCB was designed to interface with the audio codec and relays the amplified microphone signals to the analog inputs of the codec. h) HiFiBerry DAC+ ADC pro audio codec i) Raspberry Pi 4.

Comment: *No code or data provided for review.*

Response: We have now uploaded the source code.

Answers to Reviewers' Comments

Reviewer #1 (Remarks to the Author):

My comments have been incorporated into the revised version, and I recommend the manuscript for publication.

Answer: We are grateful for this comment.

Reviewer #3 (Remarks to the Author):

I would like to sincerely thank the authors for their thoughtful and thorough engagement with the reviewer comments. It is clear that considerable care was taken in addressing the feedback, and I appreciate the effort put into both the detailed responses and the revisions to the manuscript. The changes made have not only clarified several key points but have also strengthened the overall quality and coherence of the paper. In my view, these revisions have led to a significant improvement in the work. I am now fully satisfied with the current version and, as such, I am pleased to recommend the paper for acceptance.

Answer: We are grateful for this comment.
